# Multimodal Data–Driven Explainable Prognostic Model for Major Adverse Cardiovascular Events Prediction in Patients With Unstable Angina and Heart Failure With Preserved Ejection Fraction: Multicenter, Cross-Regional Cohort Study

**DOI:** 10.2196/78402

**Published:** 2025-12-15

**Authors:** Yijun Wang, Yaoling Wang, Yujie Luan, Menglei Hao, Wenyuan Yin, Bing Wu, Tongjian Zhu, Jiajun Zhu, Bowen Zhou, Long Tang, Jun Wang, Jinhui Wu

**Affiliations:** 1 Center of Gerontology and Geriatrics, National Clinical Research Center for Geriatrics, West China Hospital, Sichuan University, No. 37, Guoxue Lane, Wuhou District, Chengdu, Sichuan, 610041, China, 86 02885422332; 2 Electrocardiology Department, People’s Hospital of Xinjiang Uygur Autonomous Region, Urumqi, China; 3 Institute of Clinical Medicine and Department of Cardiology, Renmin Hospital, Hubei University of Medicine, Shiyan, China; 4 Department of Cardiology, Xiangyang Central Hospital, Xiangyang, China; 5 Department of Cardiology, The First Affiliated Hospital of Xinjiang Medical University, Urumchi, China; 6 Department of Cardiology, Suzhou First People's Hospital, Suzhou, China; 7 Department of Cardiology, The Affiliated Xuancheng Hospital of Wannan Medical College, Xuancheng, China; 8 Department of Cardiology, The First Affiliated Hospital of Bengbu Medical University, Bengbu, China

**Keywords:** heart failure with preserved ejection fraction, machine learning, major adverse cardiovascular events, risk stratification, unstable angina

## Abstract

**Background:**

Heart failure with preserved ejection fraction (HFpEF) and unstable angina (UA) often coexist in clinical practice, constituting a high-risk cardiovascular phenotype with a markedly increased incidence of major adverse cardiovascular events (MACEs). The identification of high-risk patients within this population is crucial for reducing complications, improving outcomes, and guiding clinical decision-making.

**Objective:**

This study aimed to develop and externally validate predictive models based on machine learning algorithms to estimate the risk of MACEs in patients with coexisting UA and HFpEF, and to construct an online risk calculator to support individualized prevention strategies.

**Methods:**

This multicenter cohort study included 4459 patients with both HFpEF and UA admitted to 7 hospitals across eastern, central, and western China between January 1, 2015, and December 31, 2021. Patients were divided into the derivation cohort (n=2923) and external validation cohort (n=1536) based on geographic regions. Clinical, laboratory, and imaging data were extracted from electronic medical records. Key predictors were identified using a hybrid feature selection method combining least absolute shrinkage and selection operator and Boruta algorithms. A total of 33 survival models were developed, including a variety of machine learning algorithms and survival analysis models. The model with the best concordance index (C-index) performance was deployed as a web-based risk calculator. Additionally, we assessed other performance indicators of the best-performing model, including the area under the receiver operating characteristic curve, accuracy, sensitivity, specificity, recall, *F*_1_-score, Brier scores, calibration curves, and decision curve analysis.

**Results:**

Using a combination of the least absolute shrinkage and selection operator regression and the Boruta algorithm, 7 key predictors were identified: diabetes mellitus, blood platelet count, triglyceride, systemic inflammatory response index, triglyceride-glucose–BMI, N-terminal pro-brain natriuretic peptide, and atherogenic index of plasma. The surv.xgboost.cox model was used to predict MACEs in patients with UA and HFpEF due to its superior C-index. The model demonstrated the following performance metrics in the external validation cohort: a C-index of 0.788; cumulative/dynamic area under the curve of 0.81; and area under the curve values at 20, 30, and 40 months of 0.809 (95% CI 0.745‐0.873), 0.784 (95% CI 0.745‐0.824), and 0.807 (95% CI 0.776‐0.838), respectively. The model exhibited satisfactory calibration and clinical utility in predicting 40-month MACEs. Model interpretability was enhanced using Shapley Additive Explanations for survival analysis to provide global and individual explanations. Furthermore, we converted the surv.xgboost.cox-based model into a publicly available tool for predicting 40-month MACEs, providing estimated probabilities based on the predictive indicators entered.

**Conclusions:**

We developed a surv.xgboost.cox-based predictive model for MACEs in patients with the dual phenotype of HFpEF and UA. We implemented this model as a web-based calculator to facilitate clinical application.

## Introduction

Heart failure with preserved ejection fraction (HFpEF) is a complex clinical syndrome that accounts for nearly half of all heart failure (HF) cases and is distinguished by heterogeneity in comparison to heart failure with reduced ejection fraction (HFrEF) [1,2]. Emerging epidemiological evidence highlights a significant link between HFpEF and unstable angina (UA), with cohort studies indicating a large population and a 1.3- to 3.84-fold higher risk of major adverse cardiovascular events (MACEs) in patients with this dual diagnosis compared to those with HFpEF alone [3-7]. A previous study reported that patients with HFpEF were at a higher risk of hospitalization for UA than those with HFrEF [5]. These findings indicate that the long-term prognosis for patients with UA and HFpEF is particularly concerning, especially for those with residual risk who receive suboptimal therapeutic management. Notably, the absence of validated prognostic tools that account for the underlying interactions between HFpEF-related myocardial remodeling and UA-specific ischemic burden represents a critical knowledge gap.

Both HFpEF and UA frequently share common risk factors and pathophysiological mechanisms, including systemic inflammation and metabolic abnormalities, which may contribute to their frequent coexistence and worse prognosis when present together [8-10]. This highlights the potential utility of metabolic inflammation biomarkers in risk assessment. Clinically, a comprehensive panel of laboratory tests, including routine blood tests, biochemical tests, and coagulation tests, is typically included in a general health examination. Several routine clinical laboratory indicators, including the systemic inflammation response index (SIRI), neutrophil-to-lymphocyte ratio (NLR), and the triglyceride-glucose (TyG) index, serve as markers of inflammation or metabolic status and have demonstrated prognostic significance for cardiovascular disease [11-16]. These routine clinical laboratory tests offer distinct advantages as biomarkers for coexisting HFpEF and UA due to their low cost, accessibility, consistency, and widespread use in primary health care settings.

Despite the potential of routine laboratory tests as predictive biomarkers, no single test currently has sufficient sensitivity or specificity to detect concurrent HFpEF and UA in individuals. Machine learning (ML) can assist by integrating data from multiple tests to improve risk assessment for cardiovascular diseases [16]. Technological advancements, including enhanced data storage capacity, increased computing power, and improved algorithms, enable ML to process clinically meaningful information from multidimensional clinical data. Integrating laboratory test results with artificial intelligence techniques can improve disease characterization [17].

Currently, therapeutic paradigms are restricted by diagnostic silos, and the European Society of Cardiology or American Heart Association guidelines provide limited guidance on managing this high-risk overlap population [18-20]. This highlights the need to develop accessible, efficient, and accurate predictive models to explain the complex interactions between HFpEF and UA while identifying potential risk factors. Therefore, this study aimed to provide clinical decision support for the long-term management of patients with coexisting HFpEF and UA by utilizing multimodal data from 6 centers and employing ML methods to identify MACEs risk factors and establish a longitudinal prognostic model while validating its effectiveness.

## Methods

### Study Population

This study analyzed data from the multicenter cohort, which comprises patients from 7 hospitals across China: the First Affiliated Hospital of Bengbu Medical University (Bengbu, China), the First Affiliated Hospital of Xinjiang Medical University (Urumqi, China), the People’s Hospital of Xinjiang Uygur Autonomous Region (Urumqi, China), Xiangyang Central Hospital (Xiangyang, China), Renmin Hospital, Hubei University of Medicine (Shiyan, China), the Affiliated Xuancheng Hospital of Wannan Medical College (Xuancheng, China), and Suzhou First People’s Hospital (Suzhou, China). This multicenter, cross-regional cohort study included over 10,142 cases primarily diagnosed with acute coronary syndrome and HF between January 1, 2015, and December 31, 2021, with a follow-up period of 40 months. All patients included in the cohort underwent coronary angiography during their index hospitalization to confirm the diagnosis of UA. We included 4459 patients with UA and HFpEF after data cleansing and the application of the inclusion criteria. All procedures involving human participants followed the ethical standards of the institutional research committees, the Declaration of Helsinki, and all applicable Chinese laws and regulations. The study adhered to the guidelines of the Transparent Reporting of a Multivariable Prediction Model for Individual Prognosis or Diagnosis and AI statement [21].

The primary inclusion criteria were as follows: (1) patients aged between 18 and 95 years; (2) patients with a confirmed diagnosis of UA based on coronary angiography; and (3) patients with symptoms or signs of HF, echocardiographic evidence of cardiac structural and functional abnormalities, and elevated N-terminal pro-brain natriuretic peptide (NT-proBNP) levels. We diagnosed and managed UA and HFpEF in all patients according to the established clinical guidelines [18-20]. The key exclusion criteria included the following: (1) patients with left ventricular ejection fraction <50%; (2) patients diagnosed with cancer; (3) patients with autoimmune diseases; and (4) patients who refused to participate in follow-ups. Table S1 in Multimedia Appendix 1 provides the detailed inclusion and exclusion criteria.

### Data Preprocessing

All electronic health record system variables were evaluated during the data processing phase. We prioritized comprehensive data collection by exclusively utilizing data derived from routine clinical examinations, thereby mitigating potential issues related to missing data and enhancing the integrity and reliability of our dataset. Ultimately, 72 variables, including demographic characteristics, laboratory examination, and imaging data, were included in our analysis.

Given the shared metabolic and inflammatory pathways between HFpEF and UA, we calculated and integrated several validated composite indices that indicate inflammation or metabolic conditions [22,23]. These indices include the TyG, TyG-BMI, triglyceride and high-density lipoprotein cholesterol (HDL-C) ratio (TG/HDL-C), atherogenic index of plasma (AIP), SIRI, systemic immune-inflammation index (SII), NLR, and platelet-to-lymphocyte ratio.

TyG = ln(Fasting Triglycerides (mg/dL) × Fasting Glucose (mg/dL)/2)

TyG-BMI = TyG × BMI (kg/m²)

TG/HDL-C = Triglycerides (mg/dL)/HDL-C (mg/dL)

AIP = log₁₀ (TG/HDL-C)

SIRI = Neutrophil count × Monocyte count/Lymphocyte count

SII = Platelet count × Neutrophil count/Lymphocyte count

NLR = Neutrophil/Lymphocyte ratio

PLR = Platelet/Lymphocyte ratio

### Coronary Angiography

All patients underwent coronary angiography. Coronary angiography was performed in the cardiac catheterization laboratories by experienced interventional cardiologists at each medical facility. The angiographic findings, including the presence and severity of coronary artery stenosis, were independently assessed and documented by a minimum of 2 experienced interventional cardiologists.

### Feature Selection

The least absolute shrinkage and selection operator (LASSO) regression is a linear modeling approach employed for regression analysis. It introduces a regularization term into the loss function to limit the magnitude of regression coefficients, thereby achieving variable selection and regularization. This method not only improves the model but also enhances its predictive accuracy. The LASSO regression is effective in addressing multicollinearity and exhibits robust generalization performance [24].

The Boruta algorithm is a feature selection method that employs grounded random forests. It automatically identifies and selects features most relevant to the target variable, enhancing the performance of the predictive model. The Boruta algorithm excels at managing nonlinear relationships and high-dimensional data, making it ideal for large-scale datasets while reducing the risk of overfitting [25].

We implemented a robust hybrid feature selection strategy that integrates the complementary strengths of the LASSO regression and Boruta algorithms to identify the most clinically significant predictors. The implementation of a rigorous dual-algorithm selection process conferred several methodological advantages: (1) LASSO effectively addressed multicollinearity among predictors and promoted model parsimony, (2) Boruta’s permutation-based importance testing validated the statistical significance of each selected feature, and (3) the consensus approach between the algorithms augmented the stability and reliability of the final predictor set by prioritizing variables consistently identified as significant across different mathematical frameworks. Multicollinearity was assessed using variance inflation factor analysis for all candidate predictors [26]. Variance inflation factor values exceeding 5 were considered concerning for multicollinearity, while values >10 indicated severe multicollinearity requiring intervention.

### Model Development

To ensure robust model development and assessment, the study cohort was geographically divided into the derivation cohort and the external validation cohort. The derivation cohort consisted of data from 3 centers in Anhui Province, China (n=2923; datasets from the First Affiliated Hospital of Bengbu Medical University, the Affiliated Xuancheng Hospital of Wannan Medical College, and Suzhou First People’s Hospital), while the external validation cohort included data from 4 additional centers (n=1536; datasets from the First Affiliated Hospital of Xinjiang Medical University; the People’s Hospital of Xinjiang Uygur Autonomous Region; Xiangyang Central Hospital; and Renmin Hospital, Hubei University of Medicine). The primary objective of the derivation cohort was to develop the ML models, whereas the external validation cohort was employed to evaluate the predictive performance of the models. A total of 33 survival models were employed for model construction, including *Accelerated Oblique Random Forests (surv.aorsf), eXtreme Gradient Boosting Cox Survival Learner* (surv.xgboost.cox), *Survival Gradient Boosting Machine Learner* (surv.gbm), *Gradient Boosting with Regression Trees Survival Learner* (surv.blackboost), *Survival Bayesian Additive Regression Trees Learner* (surv.bart), *Extreme Gradient Boosting AFT Survival Learner* (surv.xgboost.aft), *Survival Conditional Random Forest Learner* (surv.cforest), *Ranger Survival Learner* (surv.ranger), *Survival Random Forest SRC Learner* (surv.rfsrc), *Survival Fully Parametric Learner* (surv.parametric), *Survival Cox Model with Likelihood-Based Boosting Learner* (surv.coxboost), *Cox Proportional Hazards Survival Learner* (surv.coxph), *Survival L1 and L2 Penalized Cox Learner* (surv.penalized), *GLM with Elastic Net Regularization Survival Learner* (surv.glmnet), *Survival Priority Lasso Learner* (surv.priority_lasso), *Cross-Validated GLM with Elastic Net Regularization Survival Learner* (surv.cv_glmnet), *Boosted Generalized Additive Survival Learner* (surv.gamboost), *Boosted Generalized Linear Survival Learner* (surv.glmboost), *Boosted Generalized Additive Survival Learner* (surv.mboost), *Survival Cox Model with Cross-Validation Likelihood Based Boosting Learner* (surv.cv_coxboost), *Survival Flexible Parametric Spline Learner* (surv.flexible), *Survival Conditional Inference Tree Learner* (surv.ctree), *Survival Logistic-Hazard Learner* (surv.loghaz), *Rpart Survival Trees Survival Learner* (surv.rpart), *Survival Cox-Time Learner* (surv.coxtime), *Survival DeepSurv Learner* (surv.deepsurv), *Survival Support Vector Machine Learner* (surv.svm), *Survival Akritas Estimator Learner* (surv.akritas), *Survival DeepHit Learner* (surv.deephit), *Kaplan-Meier Estimator Survival Learner* (surv.kaplan), *Survival Nelson-Aalen Estimator Learner* (surv.nelson), *Survival PC-Hazard Learner* (surv.pchazard), and *Survival DNNSurv Learner* (surv.dnnsurv). To optimize model performance, we performed hyperparameter optimization using Grid Search by selecting the optimal hyperparameters for each algorithm.

### Model Evaluation

To select the best model, we used the concordance index (C-index) as the primary evaluation metric. The C-index, a widely used measure in survival analysis, quantifies the concordance between predicted risk scores and actual outcomes, with values ranging from 0 to 1. A higher C-index indicates better predictive accuracy. The model with the highest C-index in the external validation cohort was chosen for further analysis. The time-dependent area under the curve (AUC) of the receiver operating characteristic curve was employed to compare the model’s prediction accuracy and discrimination ability. Additionally, calibration curves were plotted to assess the agreement between predicted and actual MACEs incidence at 20-, 30-, and 40-month. Time-dependent calibration curves were used to reflect the model’s calibration across the entire time range. Decision curve analysis was performed to evaluate the clinical benefit of the model at 20, 30, and 40 months, helping to determine its clinical utility. We evaluated other key performance metrics for the best-performing model, including the integrated cumulative/dynamic area under the curve (C/D AUC) and Brier scores. The cut-off value of 40 months was determined by maximizing the Youden index in the derivation cohort, which corresponds to the predicted 40-month MACE probability (risk) and serves as the threshold for model interpretation. This cutoff value was then applied in external validation to assess the model’s performance. The binary classification metrics evaluated included accuracy, recall (sensitivity), specificity, positive predictive value (PPV), negative predictive value (NPV), and *F*_1_-score at the 20-, 30-, and 40-month time points, as detailed in Table S3 in Multimedia Appendix 1.

### Model Interpretation

We utilized the Survex interpreter to provide both global and local interpretations of model predictions, thereby enhancing the interpretability of survival analysis models [27].

For global explanations, we employed time-dependent Shapley Additive Explanations for survival analysis (SurvSHAP(t)), time-dependent feature importance curves, and partial dependence survival profiles (PDPs). SurvSHAP(t) is an extension of the SHAP method designed specifically for survival models, allowing for the decomposition of survival predictions and visualizing the contribution of each feature to individual predictions over time. The time-dependent feature importance plots provide insights into the evolution of feature importance over time, capturing the dynamic impact of features on survival predictions. Additionally, PDPs were utilized to assess the marginal effects of individual features on the model’s predictions. PDPs facilitate the understanding of the relationship between the target and features, thereby enhancing the comprehension of how individual features influence survival outcomes. For local explanations, we applied SurvSHAP(t) and local interpretable model-agnostic explanations for survival models. Local interpretable model-agnostic explanations for survival models approximates the survival model locally by fitting a proportional hazards model to the neighborhood of each observation, providing a patient-specific explanation based on the model’s coefficients.

### Development of a Web-Based Calculator

Based on the optimal model, a web-based calculator was developed using the Shiny application framework to predict the 40-month risk of MACEs in these patients. To improve user accessibility and facilitate seamless interaction, we designed a calculator with 3 distinct panels: (1) a parameter selection and input panel, (2) a results display panel for the predicted 40-month MACEs risk, and (3) an interpretability panel that utilizes SurvSHAP(t) values to explain the predictions of the model.

### Clinical End Points

Clinical follow-up was performed through outpatient consultations or telephone interviews. The average follow-up duration for the study population was 37.9 months. The primary clinical endpoint of the study was the occurrence of MACEs within a 40-month period, which included a composite outcome of cardiac death, non-fatal myocardial infarction, and unplanned revascularization following hospital discharge, all of which occur post-discharge.

### Ethical Considerations

This study was approved by the institutional review boards of the following institutions: the First Affiliated Hospital of Bengbu Medical University ([2023]KY049X01); the First Affiliated Hospital of Xinjiang Medical University (K202507-02); the Affiliated Xuancheng Hospital of Wannan Medical College (No. 2023‐1w004-01); the People’s Hospital of Xinjiang Uygur Autonomous Region (KY20230111001); Xiangyang Central Hospital (2023‐023); Renmin Hospital, Hubei University of Medicine (SYRMYY-2024‐017); and Suzhou First People’s Hospital (SZYYLLKY2024015). The study was registered in the Chinese Clinical Trial Registry with the registration No. ChiCTR2400080282. Given the retrospective design of the study, the need to obtain informed consent from eligible patients was waived by the Ethics Committees.

### Statistical Analysis

Categorical variables are summarized using descriptive statistics, including frequencies and percentages. Continuous variables are presented as medians with interquartile ranges (IQRs) or means (SDs) based on their distribution characteristics. Categorical data are presented as counts and percentages, while medians with IQRs for non-normal distributions represent continuous data and means with SDs for normal distributions. The Mann-Whitney *U* test was employed to compare continuous variables between the training and validation groups for non-normally distributed data. Categorical variables were analyzed using the Fisher exact test or the *χ*² test, as appropriate. All statistical analyses and modeling procedures were performed using R software (version 4.2.2) and Python software (version 3.11.4). A 2-sided *P* value of <.05 was considered statistically significant.

## Results

### Patient Characteristics

**Figure 1.**
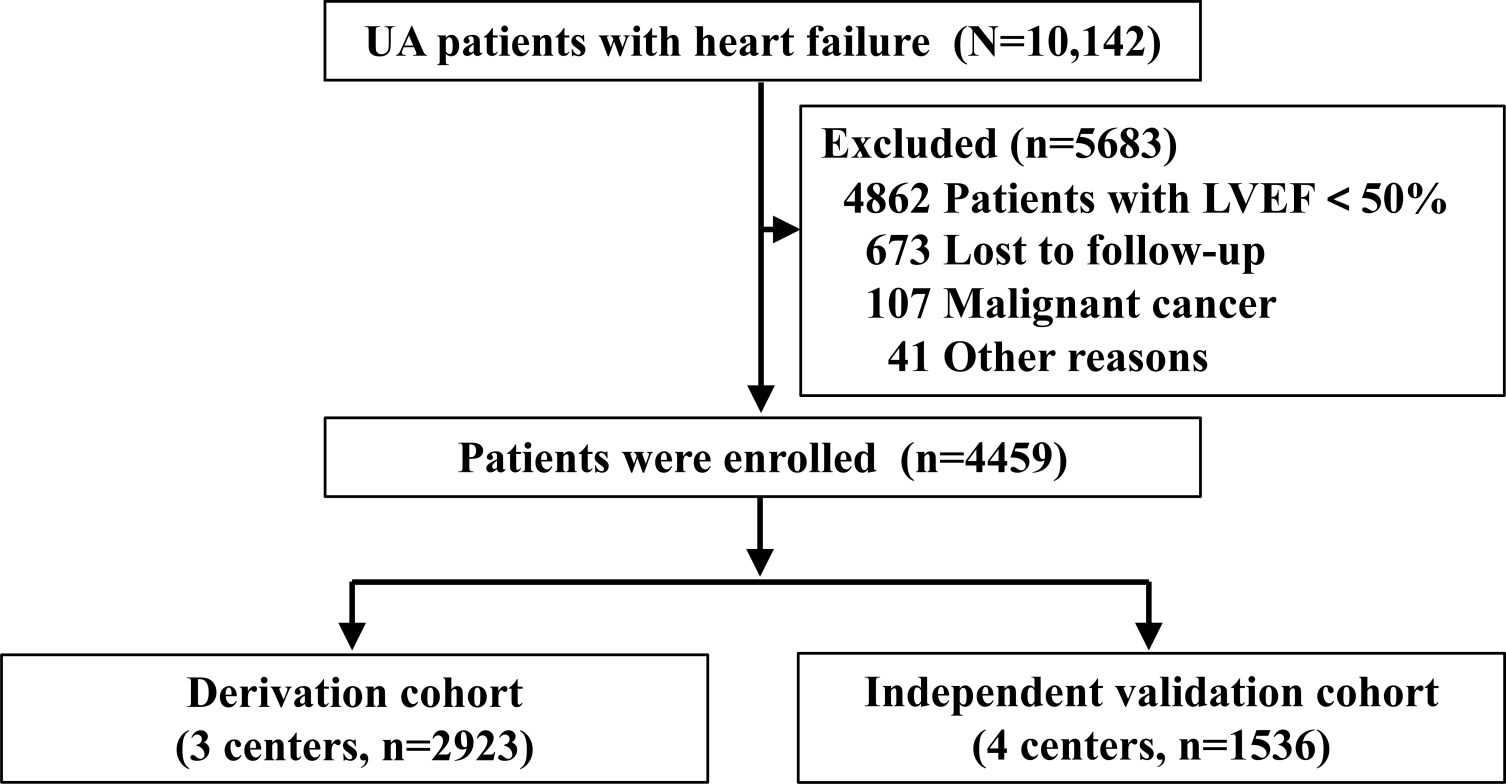
Flowchart outlining patient enrollment. LVEF: left ventricular ejection fraction; UA: unstable angina.

We enrolled 4459 patients with UA and HFpEF in our study, as determined by the inclusion and exclusion criteria. Figure 1 illustrates the flowchart that summarizes patient enrollment and study design. The cohort included 57.73% (n=2574) male participants, with an average age of 67.00 years (IQR 58.00‐75.00) for all the evaluated patients. We recorded 610 MACEs (n=610, 13.68%) over a median follow-up period of 37.9 months. The incidence rates for the derivation and external validation cohorts were similar (n=416, 14.23%, and n=194, 12.63%, respectively). Table 1 summarizes the baseline characteristics of patients with UA and HFpEF.

**Table 1.** Baseline patient characteristics and MACEs^a^ (clinical outcome) in the derivation and external validation cohorts.

	All cohort (N=4459)	Derivation cohort (n=2923)	Validation cohort (n=1536)	*P* value
Male sex, n (%)	2574 (57.73)	1684 (57.61)	890 (57.94)	.86
Age (y), median (IQR)	67 (58 to 75)	68 (57 to 75)	67 (59 to 74)	.88
BMI (kg/m^2^), median (IQR)	23.77 (20.86 to 27.14)	23.63 (20.57 to 27.24)	23.91 (21.27 to 27.04)	.10
Hypertension, n (%)	2802 (62.84)	1856 (63.50)	946 (61.59)	.22
Diabetes mellitus, n (%)	1450 (32.52)	973 (33.29)	477 (31.05)	.14
Current smoking, n (%)	1405 (31.51)	904 (30.93)	501 (32.62)	.26
Current drinking, n (%)	873 (19.58)	561 (19.19)	312 (20.31)	.39
Family history of CAD^b^, n (%)	246 (5.52)	156 (5.34)	90 (5.86)	.51
Previous myocardial infarction, n (%)	274 (6.14)	171 (5.85)	103 (6.71)	.29
Previous PCI^c^, n (%)	1043 (23.39)	680 (23.26)	363 (23.63)	.81
Previous stroke, n (%)	373 (8.37)	239 (8.18)	134 (8.72)	.57
Use of aspirin, n (%)	3214 (72.08)	2084 (71.30)	1130 (73.57)	.12
Use of clopidogrel, n (%)	1245 (27.92)	839 (28.70)	406 (26.43)	.12
Use of β receptor blockers, n (%)	2129 (47.75)	1440 (49.26)	689 (44.86)	.006
Use of ACEI^d^, n (%)	631 (14.15)	426 (14.57)	205 (13.35)	.28
Use of ARB^e^, n (%)	873 (19.58)	672 (22.99)	201 (13.09)	<.001
Use of CCB^f^, n (%)	1029 (23.08)	670 (22.92)	359 (23.37)	.76
Use of sacubitril valsartan, n (%)	789 (17.69)	717 (24.53)	72 (4.69)	<.001
SBP^g^ (mm Hg), median (IQR)	139 (128 to 150)	135 (126 to 154)	142 (134 to 148)	<.001
DBP^h^ (mm Hg), median (IQR)	84 (76 to 91)	82 (74 to 93)	86 (76 to 90)	<.001
Heart rate (times per minute), median (IQR)	75 (70 to 81)	78 (71 to 82)	70 (70 to 76)	<.001
White blood cell count (10^9^/L), median (IQR)	6.18 (5.13 to 7.60)	6.20 (5.14 to 7.58)	6.12 (5.11 to 7.62)	.82
Neutrophil count (10^9^/L), median (IQR)	3.99 (3.13 to 5.20)	3.98 (3.15 to 5.18)	4.02 (3.11 to 5.23)	.95
Lymphocyte count (10^9^/L), median (IQR)	1.53 (1.17 to 2.00)	1.53 (1.19 to 1.98)	1.56 (1.12 to 2.04)	.60
Monocyte count (10^9^/L), median (IQR)	0.39 (0.27 to 0.56)	0.40 (0.28 to 0.55)	0.36 (0.27 to 0.58)	.91
Blood platelet count (10^9^/L), median (IQR)	191 (156 to 236)	191 (154 to 236)	193 (159 to 234)	.18
NLR^i^, median (IQR)	2.58 (1.86 to 3.90)	2.58 (1.85 to 3.85)	2.59 (1.87 to 4.04)	.45
PLR^j^, median (IQR)	123.23 (92.38 to 169.23)	121.80 (92.48 to 166.91)	126.40 (92.18 to 173.03)	.07
SII^k^, median (IQR)	492.36 (329.04 to 777.42)	488.63 (331.47 to 758.52)	502.54 (326.11 to 809.26)	.19
SIRI^l^, median (IQR)	0.98 (0.61 to 1.67)	0.98 (0.61 to 1.62)	0.95 (0.61 to 1.85)	.11
Mean platelet volume, median (IQR)	10.70 (9.96 to 11.70)	10.70 (9.96 to 11.70)	10.70 (10 to 11.70)	.25
Thrombocytocrit (%), median (IQR)	0.22 (0.18 to 0.27)	0.21 (0.18 to 0.26)	0.22 (0.17 to 0.28)	.07
Mean platelet width (%), median (IQR)	16.20 (14.30 to 18.50)	16.40 (14.90 to 18.40)	15.60 (12.40 to 18.60)	<.001
Hemoglobin (g/L), median (IQR)	131 (118 to 144)	131 (120 to 144)	130 (116 to 146)	.47
Creatinine (μmol/L), median (IQR)	75.00 (63.00 to 90.40)	72.00 (60.00 to 89.00)	80.00 (68.70 to 92.00)	<.001
Uric acid (μmol/L), median (IQR)	335.40 (278.00 to 412.00)	336.50 (274.00 to 420.00)	333.00 (284.00 to 402.25)	.57
Fasting blood glucose (mmol/L), median (IQR)	5.71 (4.99 to 7.23)	5.72 (5.03 to 7.16)	5.67 (4.90 to 7.29)	.19
NT-proBNP^m^ (ng/L), median (IQR)	345.00 (195.00 to 569.22)	345.66 (195.00 to 571.58)	343.52 (194.90 to 564.68)	.33
Triglyceride (mmol/L), median (IQR)	1.36 (0.94 to 1.83)	1.34 (0.95 to 1.81)	1.39 (0.90 to 1.88)	.71
Total cholesterol (μmol/L), median (IQR)	4.32 (3.84 to 5.17)	4.32 (3.82 to 5.16)	4.32 (3.88 to 5.18)	.78
HDL-C^n^ (mmol/L), median (IQR)	1.08 (0.91 to 1.29)	1.07 (0.91 to 1.28)	1.09 (0.92 to 1.30)	.19
LDL-C^o^ (mmol/L), median (IQR)	2.41 (1.83 to 3.05)	2.41 (1.85 to 3.02)	2.42 (1.80 to 3.12)	.44
TyG^p^, median (IQR)	8.78 (8.36 to 9.20)	8.79 (8.38 to 9.18)	8.77 (8.32 to 9.25)	.56
TyG-BMI, median (IQR)	208.76 (181.14 to 241.38)	207.41 (178.94 to 242.92)	210.89 (185.06 to 238.43)	.16
AIP^q^, median (IQR)	0.10 (–0.09 to 0.27)	0.10 (–0.08 to 0.27)	0.09 (–0.11 to 0.28)	.30
Lipoprotein a (mg/L), median (IQR)	168.00 (67.00 to 363.00)	184.00 (62.15 to 361.65)	155.82 (71.00 to 367.00)	.17
Apolipoprotein AI (g/L), median (IQR)	1.11 (0.91 to 1.35)	1.10 (0.91 to 1.35)	1.11 (0.91 to 1.34)	.61
Apolipoprotein B (g/L), median (IQR)	0.94 (0.73 to 1.06)	0.94 (0.71 to 1.08)	0.94 (0.77 to 1.03)	.81
Cystatin C (mg/L), median (IQR)	0.93 (0.79 to 1.10)	0.90 (0.76 to 1.11)	0.97 (0.84 to 1.09)	<.001
Total bilirubin (μmol/L), median (IQR)	13.30 (9.80 to 17.60)	13.50 (9.80 to 17.84)	13.20 (9.70 to 17.00)	.10
Direct bilirubin (μmol/L), median (IQR)	4.40 (3.00 to 7.00)	4.40 (2.90 to 7.30)	4.40 (3.10 to 6.70)	.61
Indirect bilirubin (μmol/L), median (IQR)	7.90 (5.50 to 11.00)	7.80 (5.36 to 11.40)	8.10 (5.90 to 10.61)	.20
ALT^r^ (U/L), median (IQR)	20.90 (15.00 to 31.00)	21.00 (15.00 to 30.20)	20.00 (15.00 to 32.00)	.35
AST^s^ (U/L), median (IQR)	22.00 (17.40 to 31.00)	22.00 (17.80 to 30.00)	23.00 (17.00 to 32.00)	.91
Gamma-glutamyl transferase (U/L), median (IQR)	26.00 (18.00 to 37.65)	25.50 (17.50 to 38.80)	26.40 (19.00 to 36.10)	.63
ALT/AST, median (IQR)	0.93 (0.70 to 1.22)	0.93 (0.71 to 1.22)	0.92 (0.67 to 1.23)	.20
Fibrinogen (g/L), median (IQR)	2.90 (2.34 to 3.75)	2.92 (2.30 to 3.77)	2.89 (2.40 to 3.70)	.10
D-Dimer (g/L), median (IQR)	0.29 (0.23 to 0.51)	0.32 (0.25 to 0.64)	0.26 (0.22 to 0.32)	<.001
Total protein (g/L), median (IQR)	68.30 (63.30 to 73.70)	68.10 (63.00 to 73.60)	68.80 (64.00 to 73.90)	.009
Albumin (g/L), median (IQR)	40.50 (38.60 to 43.20)	40.70 (37.70 to 43.85)	40.40 (39.50 to 41.32)	.33
Globulin (g/L), median (IQR)	27.20 (23.60 to 31.50)	27.10 (23.70 to 31.30)	27.40 (23.50 to 32.20)	.30
Albumin or globulin, median (IQR)	1.50 (1.28 to 1.73)	1.50 (1.29 to 1.72)	1.51 (1.25 to 1.77)	.72
LVEF^t^ (%), median (IQR)	56 (52 to 59)	55 (51 to 60)	56 (53 to 59)	.25
LVEDD^u^ (mm), median (IQR)	49 (45 to 52)	49 (45 to 52)	49 (45 to 52)	.93
LVPWD^v^ (mm), median (IQR)	10 (9 to 11)	10 (9 to 11)	10 (9 to 11)	.63
IVSD^w^ (mm), median (IQR)	10 (9 to 11)	10.00 (8 to 11.00)	10 (9 to 11)	.24
LAD^x^ (mm), median (IQR)	35 (32 to 38)	35.00 (32 to 37)	36 (33 to 38)	<.001
Number of diseased vessels, n (%)				.01
1	2926 (65.62)	1890 (64.66)	1036 (67.45)	
2	631 (14.15)	447 (15.29)	184 (11.98)	
3	902 (20.23)	586 (20.05)	316 (20.57)	
LAD stenosis, n (%)	1599 (35.86)	1045 (35.75)	554 (36.07)	.86
LCX^y^ stenosis, n (%)	1087 (24.38)	725 (24.80)	362 (23.57)	.38
RCA^z^ stenosis, n (%)	3072 (68.89)	2008 (68.70)	1064 (69.27)	.72
MACEs, n (%)	610 (13.68)	416 (14.23)	194 (12.63)	.15
Cardiac mortality	142 (3.18)	95 (3.25)	47 (3.06)	.80
Revascularization	420 (9.42)	286 (9.78)	134 (8.72)	.27
Nonfatal myocardial infarction	101 (2.27)	63 (2.16)	38 (2.47)	.57

a MACE: major adverse cardiovascular event.

b CAD: coronary artery disease.

c PCI: percutaneous coronary intervention.

d ACEI: angiotensin-converting enzyme inhibitors.

e ARB: angiotensin receptor blockers.

f CCB: calcium channel blockers.

g SBP: systolic blood pressure.

h DBP: diastolic blood pressure.

i NLR: neutrophil-to-lymphocyte ratio.

j PLR: platelet-to-lymphocyte ratio.

k SII: systemic immune-inflammation index.

l SIRI: systemic inflammation response index.

m NT-proBNP: N-terminal pro-brain natriuretic peptide.

n HDL-C: high-density lipoprotein cholesterol.

o LDL-C: low-density lipoprotein cholesterol.

p TyG: triglyceride-glucose.

q AIP: atherogenic index of plasma.

r ALT: alanine transaminase.

s AST: aspartate transferase.

t LVEF: left ventricular ejection fraction.

u LVEDD: left ventricular end-diastolic diameter.

v LVPWD: left ventricular posterior wall thickness in diastole.

w IVSD: interventricular septum thickness in diastole.

x LAD: left anterior descending.

y LCX: left circumflex coronary artery.

z RCA: right coronary artery.

### Feature Selection in Models

In the LASSO model with 10-fold cross-validation, 17 clinical features had nonzero coefficients at the optimal Lambda value. Figure S1A,B in Multimedia Appendix 1 outlines the comprehensive screening procedure. At the optimal penalty level, the LASSO regression identified 17 significant variables: diabetes mellitus, family history of coronary artery disease, previous percutaneous coronary intervention, use of clopidogrel, use of sacubitril valsartan, heart rate, blood platelet count, SIRI, NT-proBNP, TyG, TyG-BMI, AIP, apolipoprotein AI, cystatin C, total bilirubin, indirect bilirubin, and fibrinogen.

Boruta iteratively eliminated statistically nonsignificant features and identified strongly and weakly correlated features with the output variables. After 100 iterations, the algorithm confirmed 24 significant variables: BMI, diabetes mellitus, neutrophil count, lymphocyte count, blood platelet count, NLR, platelet-to-lymphocyte ratio, SII, SIRI, creatinine, uric acid, fasting blood glucose, NT-proBNP, triglyceride, total cholesterol, HDL-C, low-density lipoprotein cholesterol, TyG, TyG-BMI, AIP, lipoprotein a, apolipoprotein B, total protein, and albumin. Figure S1C,D in Multimedia Appendix 1 depicts the changes in importance scores for each variable during Boruta’s operation and the evolution of *z* scores.

**Figure 2.**
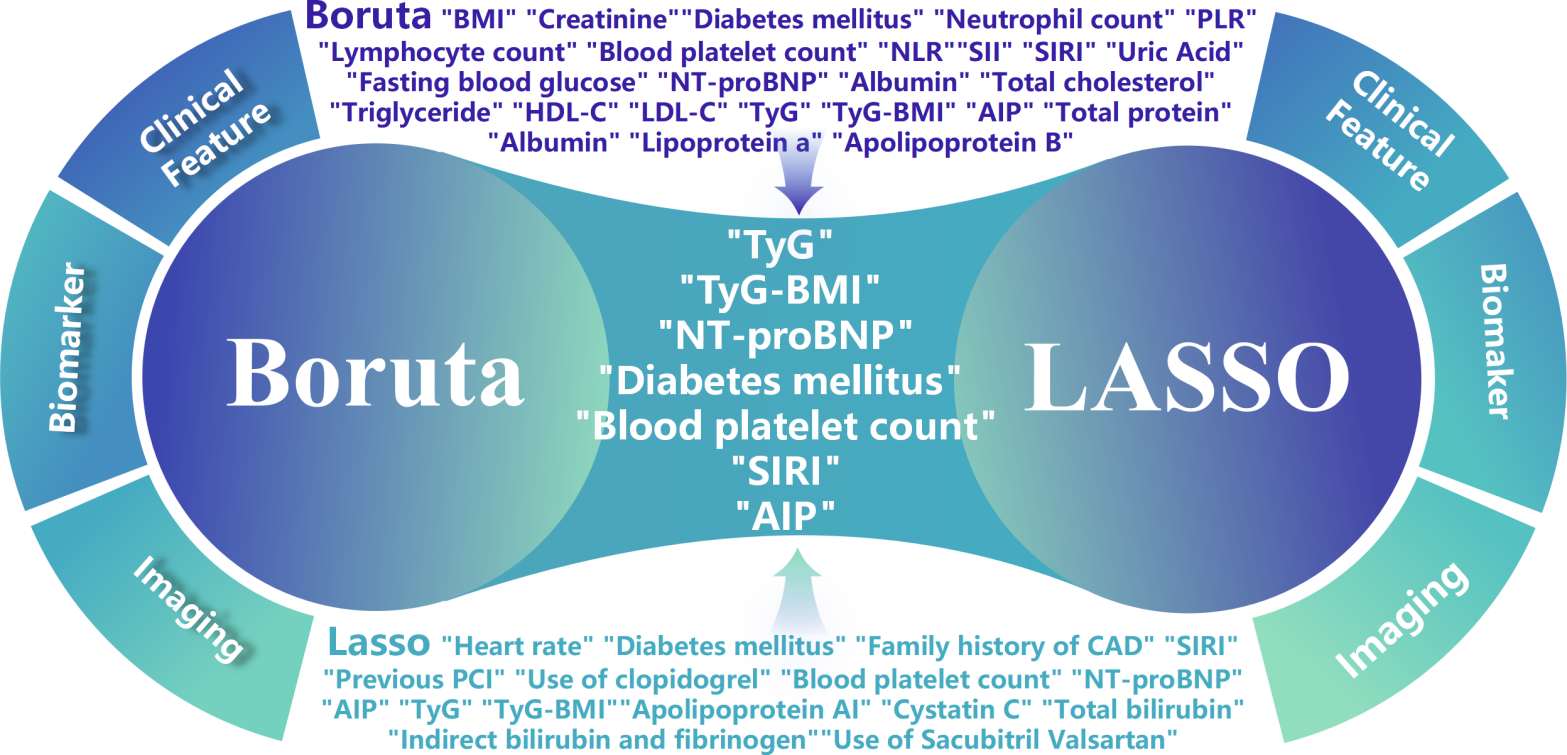
The Boruta algorithms and least absolute shrinkage and selection operator (LASSO) regression analysis identified 7 variables. AIP: atherogenic index of plasma; CAD: coronary artery disease; HDL-C: high-density lipoprotein cholesterol; LDL-C: low-density lipoprotein cholesterol; NLR: neutrophil-to-lymphocyte ratio; NT-proBNP: N-terminal pro-brain natriuretic peptide; PCI: percutaneous coronary intervention; PLR: platelet-to-lymphocyte ratio; SII: systemic immune-inflammation index; SIRI: systemic inflammatory response index; TyG: triglyceride-glucose.

The predictive variables identified by LASSO and Boruta algorithms were selected as standard variables for developing the prediction model. A total of 7 clinical variables were identified as predictive factors: diabetes mellitus, blood platelet count, SIRI, NT-proBNP, TyG, TyG-BMI, and AIP (Figure 2 and Figure S2 in Multimedia Appendix 1).

### Development and Comparison of Prediction Models

We employed 33 survival models to predict the 40-month risk of MACEs in patients with UA and HFpEF. The models included surv.aorsf, surv.xgboost.cox, surv.gbm, surv.blackboost, surv.bart, surv.xgboost.aft, surv.cforest, surv.ranger, surv.rfsrc, surv.parametric, surv.coxboost, surv.coxph, surv.penalized, surv.glmnet, surv.priority_lasso, surv.cv_glmnet, surv.gamboost, surv.glmboost, surv.mboost, surv.cv_coxboost, surv.flexible, surv.ctree, surv.loghaz, surv.rpart, surv.coxtime, surv.deepsurv, surv.svm, surv.akritas, surv.deephit, surv.kaplan, surv.nelson, surv.pchazard, and surv.dnnsurv. Following hyperparameter optimization, each model was constructed using the derivation cohort, and the optimal configurations for all 33 models are listed in Table S4 in Multimedia Appendix 1. Subsequently, the performance of the best model was assessed on the external validation cohort to determine predictive accuracy.

**Figure 3.**
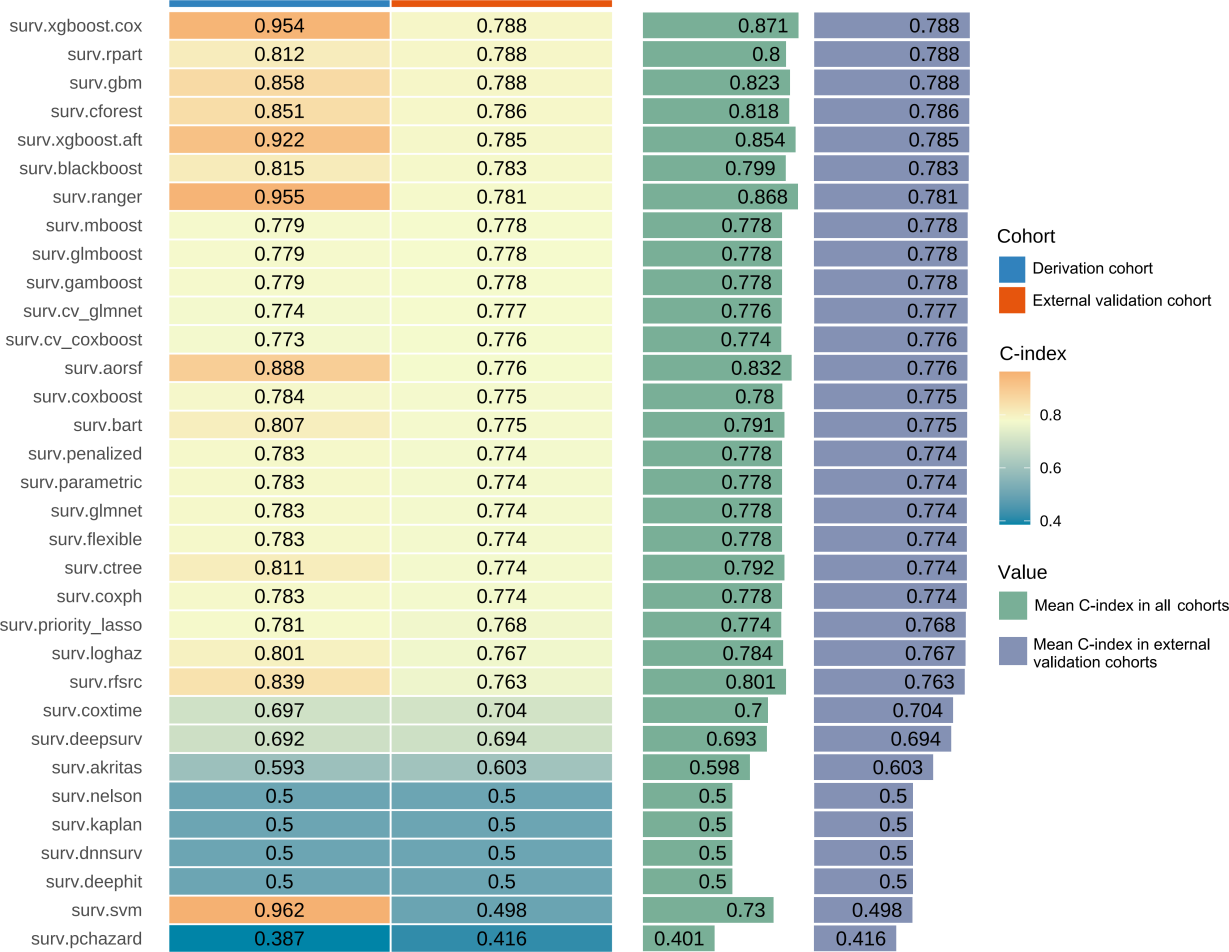
Concordance index (C-index) of 33 prognostic models in the derivation and external validation cohorts, along with the average C-index.

The performance evaluation focused on the C-index, and Figure 3 presents the average C-index for all 33 predictive models. The surv.xgboost.cox model, which is based on the linear predictor derived from the Cox proportional hazards model, exhibited the highest average C-index of 0.871, making it the most effective predictive tool among all models. In the derivation cohort, the surv.xgboost.cox model achieved a C-index of 0.954, while in the external validation cohort, the C-index was 0.788, both of which were higher than those of the other models.

**Figure 4.**
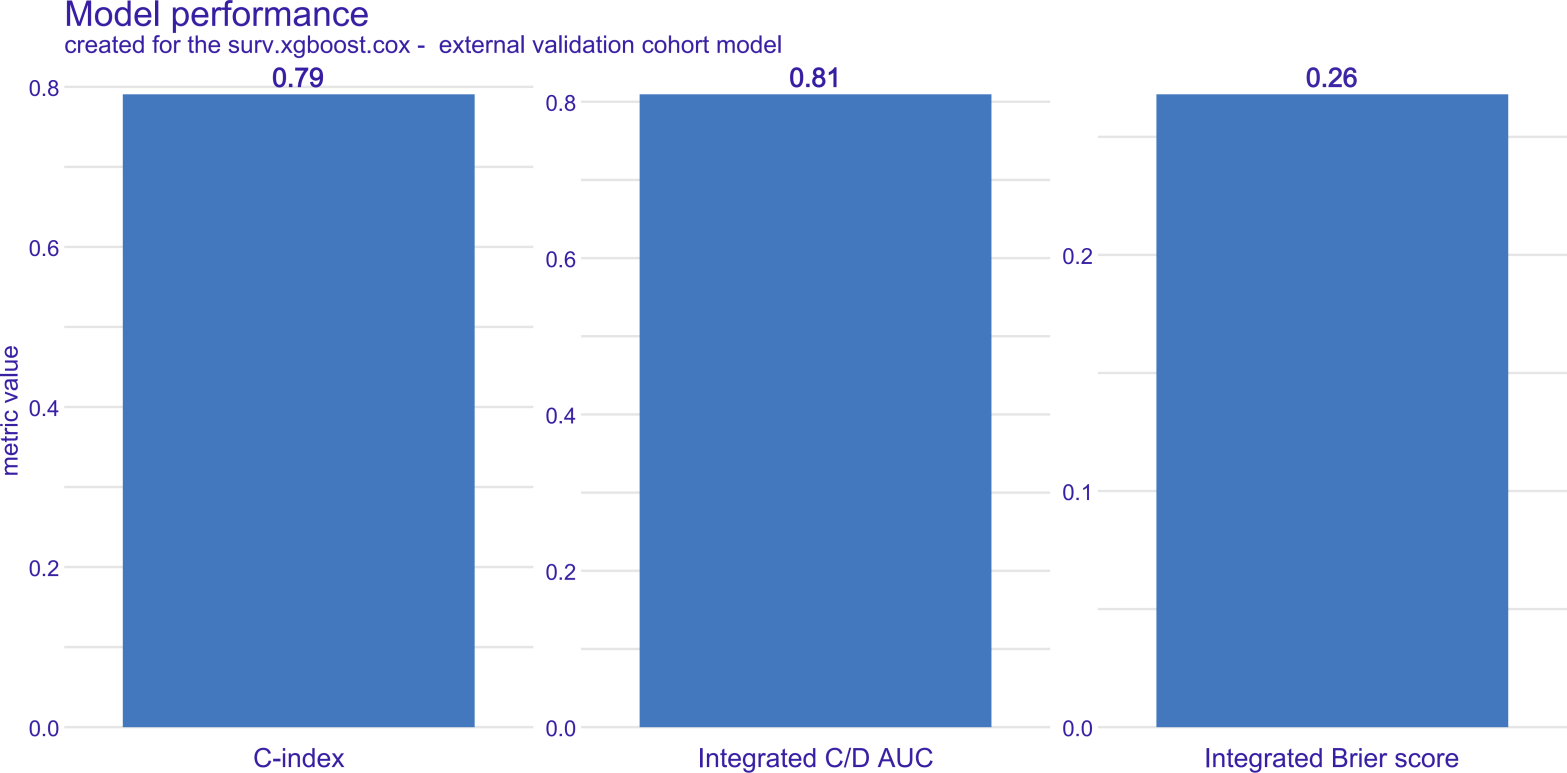
Performance evaluation of the surv.xgboost.cox model: Brier score and cumulative/dynamic area under the curve (C/D AUC) in the external validation cohorts. C-index: concordance index.

We further evaluated the Brier score and C/D AUC. In the derivation cohort, the Brier score for the surv.xgboost.cox model was 0.18, with a C/D AUC of 0.96. In the external validation cohort, the Brier score was 0.27, and the C/D AUC was 0.81 (Figure 4).

**Figure 5.**
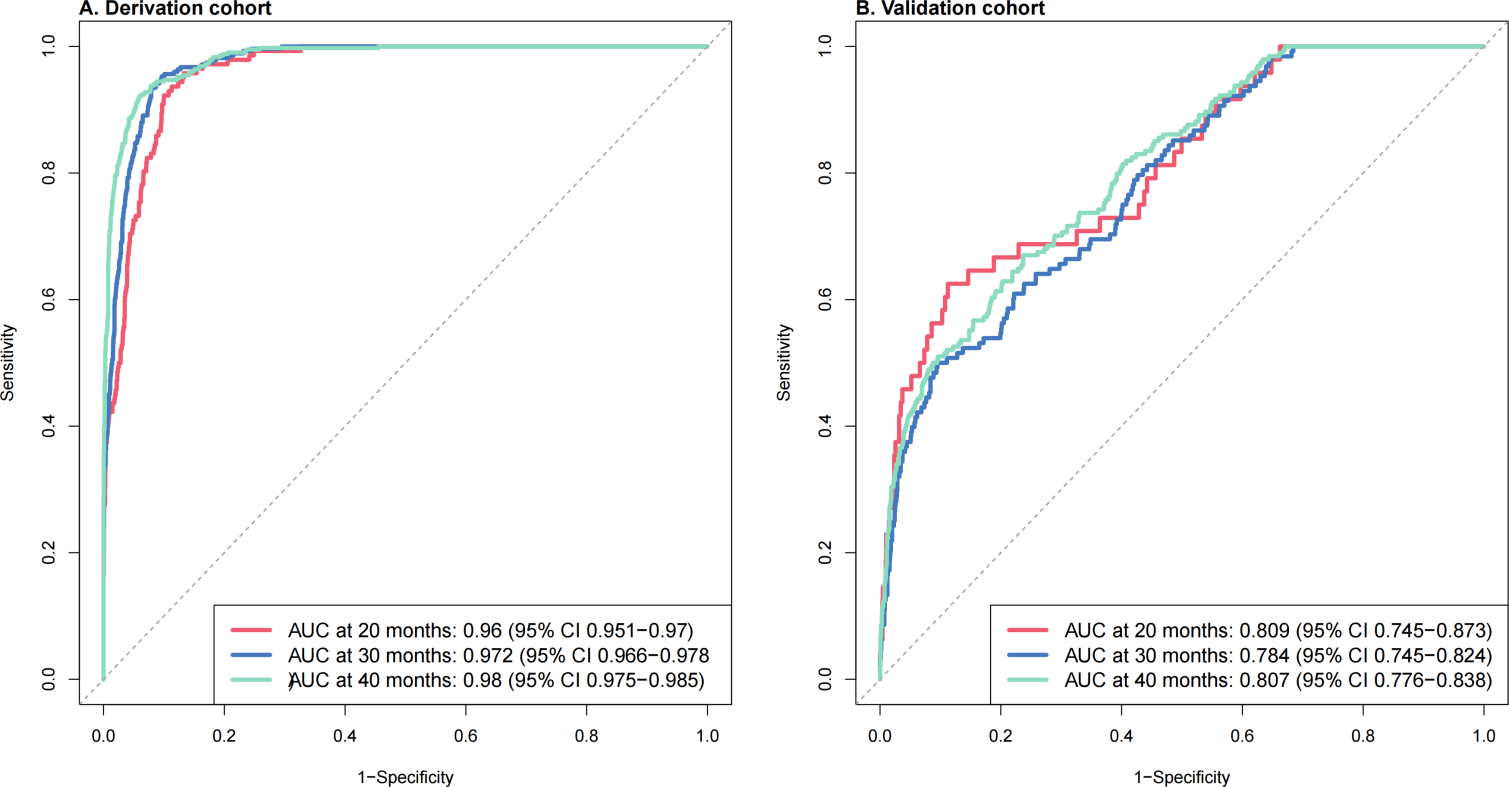
Time-dependent receiver operating characteristic (ROC) curves for the surv.xgboost.cox model at 20, 30, and 40 months in the derivation and external validation cohorts. AUC: area under the curve.

The time-dependent ROC curves demonstrated that in both the derivation cohort and external validation cohorts, the surv.xgboost.cox model exhibited strong predictive accuracy at the 20-, 30-, and 40-month time points. In the derivation cohort, the surv.xgboost.cox model achieved an AUC of 0.96 (95% CI 0.951‐0.97) at 20 months, 0.972 (95% CI 0.966‐0.978) at 30 months, and 0.98 (95% CI 0.975‐0.985) at 40 months (Figure 4A). In the external validation cohort, the surv.xgboost.cox model reached an AUC of 0.809 (95% CI 0.745‐0.873) at 20 months, 0.784 (95% CI 0.745‐0.824) at 30 months, and 0.807 (95% CI 0.776‐0.838) at 40 months (Figure 5).

**Figure 6.**
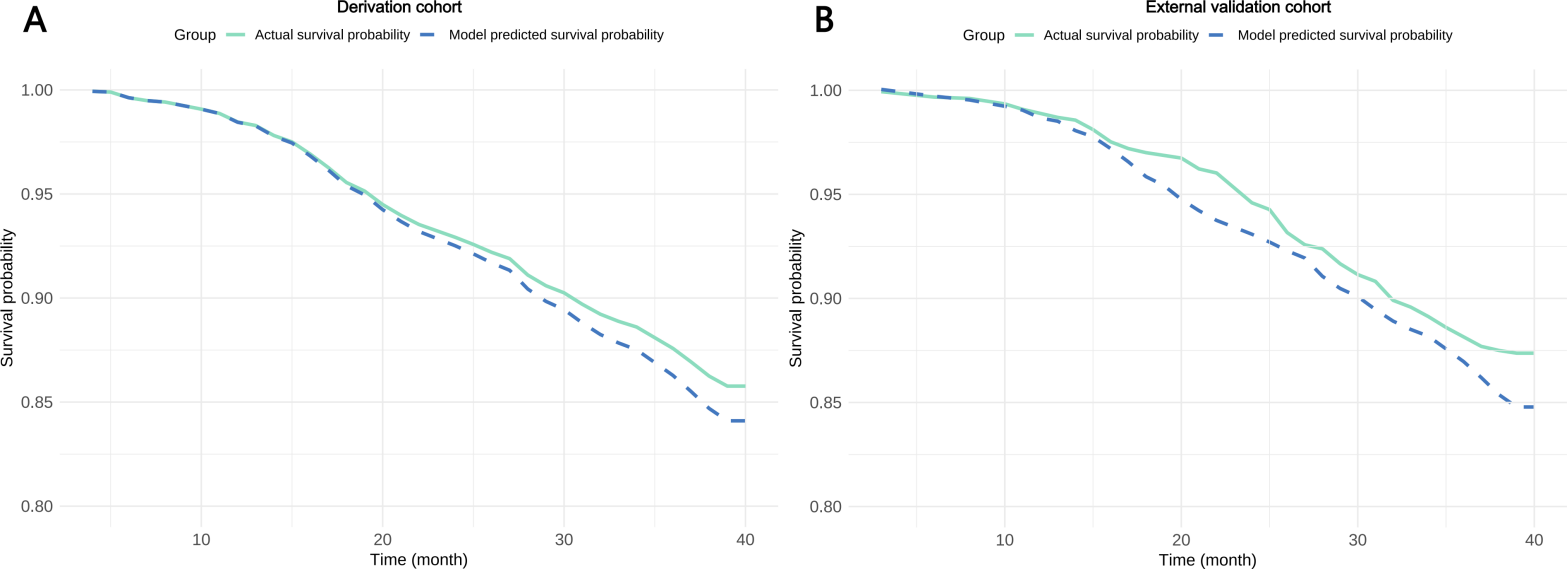
Time-dependent calibration curves for the surv.xgboost.cox model in the derivation and external validation cohorts.

The time-dependent calibration curves, along with the 20-, 30-, and 40-month calibration curves for the surv.xgboost.cox model, indicated moderate calibration performance in both the derivation cohort and the external validation cohort (Figure 6). Decision curve analysis further confirmed that the surv.xgboost.cox model demonstrated significant clinical utility (Figure S4 in Multimedia Appendix 1).

The cut-off values and the 7 predictive metrics were calculated to comprehensively assess model performance at the 20-, 30-, and 40-month time points: accuracy, recall, sensitivity, specificity, PPV, NPV, and *F*_1_-score (Tables S3 in Multimedia Appendix 1). The surv.xgboost.cox model in the external validation cohort demonstrated the following performance metrics at the 40-month time point: cut-off value of 0.225, accuracy of 0.893, recall of 0.340, specificity of 0.972, PPV of 0.641, NPV of 0.911, and *F*_1_-score of 0.444.

### Model Interpretation

**Figure 7.**
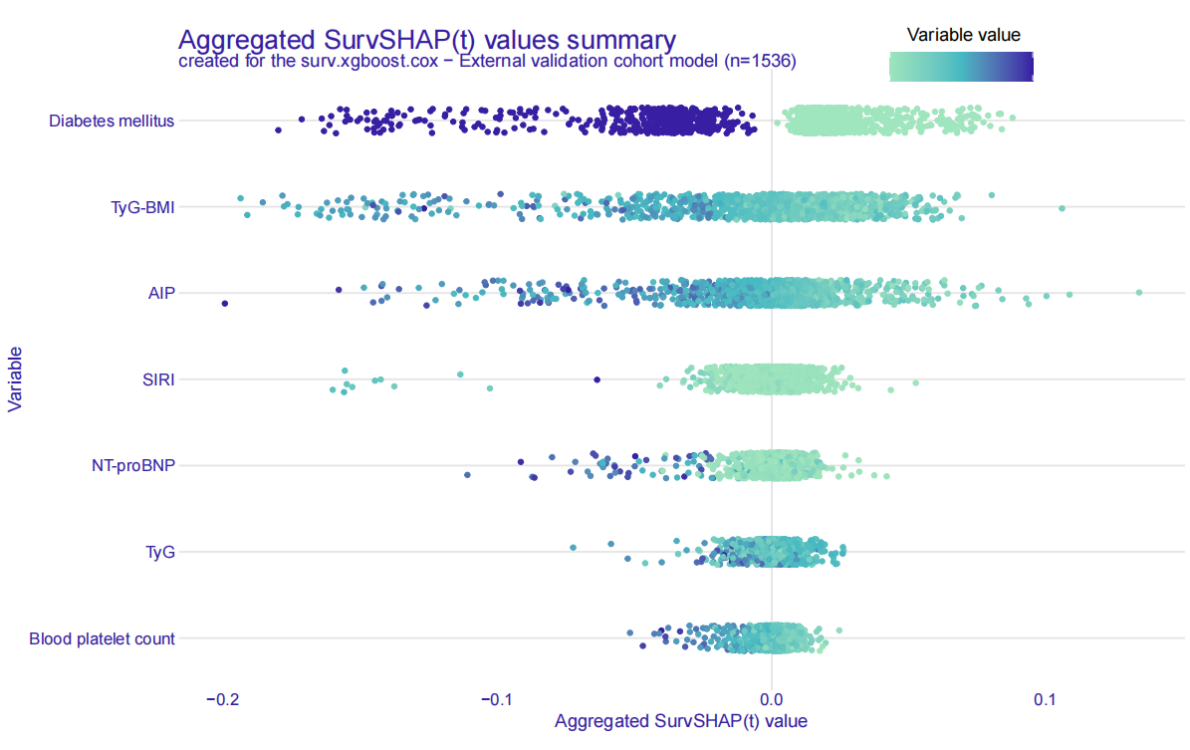
Beeswarm plot of Shapley Additive Explanations for survival analysis (SurvSHAP(t)) values, showing feature importance and the impact of predictors on the surv.xgboost.cox model’s predictions. AIP: atherogenic index of plasma; NT-proBNP: N-terminal pro-brain natriuretic peptide; SIRI: systemic inflammation response index; TyG: triglyceride-glucose.

**Figure 8.**
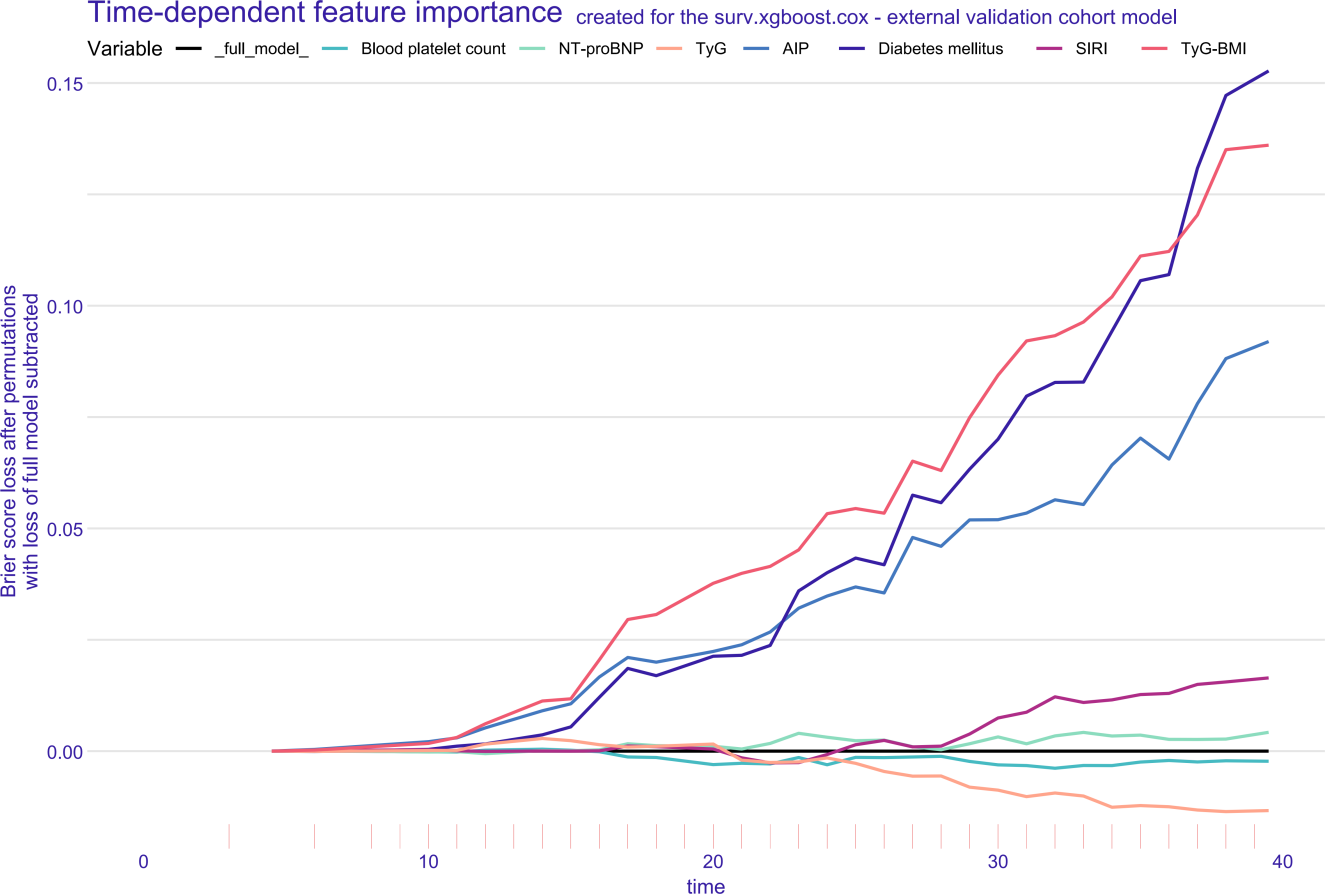
Brier score-based time-dependent feature importance plots for the surv.xgboost.cox model. AIP: atherogenic index of plasma; NT-proBNP: N-terminal pro-brain natriuretic peptide; SIRI: systemic inflammation response index; TyG: triglyceride-glucose.

SurvSHAP(t) values were used to assess the contribution of each feature to the final prediction and to interpret outcomes for individual patients. Figure 7 presents a beeswarm plot of SurvSHAP(t) values, showing the distribution from low (green) to high (blue) for each predictor, offering a global interpretation of feature importance and illustrating the overall impact of individual features on the model’s predictions across the cohort, providing insights into the global relationships between features and predicted outcomes. Additionally, SurvSHAP(t)-based univariate dependence plots illustrate the time-dependent relationships between the 7 key features and the predicted 40-month MACEs risk (Figure S7 in Multimedia Appendix 1). The SurvSHAP(t) risk score plots further highlight the impact of metabolic-inflammatory factors on the predicted 40-month MACEs risk, providing patient-specific profiles that can guide more personalized management strategies (Figure S8 in Multimedia Appendix 1). To ensure clarity and consistency, the definition and directionality of the risk score are as follows: The higher the risk score, the higher the predicted risk. Higher scores correspond to an increased risk of experiencing MACEs, while lower scores correspond to a lower risk. These profiles help clinicians better understand individualized risk factors, enhancing decision-making in the management of patients at risk for MACEs. Figure 8 presents the Brier score−based time-dependent feature importance plots, providing insights into the significance of each feature over time. Figure S6 in Multimedia Appendix 1 illustrates the partial dependence survival profiles, showing the marginal effects of feature variables on the final survival outcome.

### Application of the Model

We created an online calculator that predicts the 40-month risk of MACEs in patients with UA and HFpEF using the Shiny application framework and the optimal model. To improve usability and interpretability, we integrated Survex into the calculator for global interpretation and to provide quantitative estimates of MACE risk. Users can easily obtain prediction results by entering clinical feature data into specified text fields on the web interface (Figure S9 in Multimedia Appendix 1).

## Discussion

### Principal Findings

**Figure 9.**
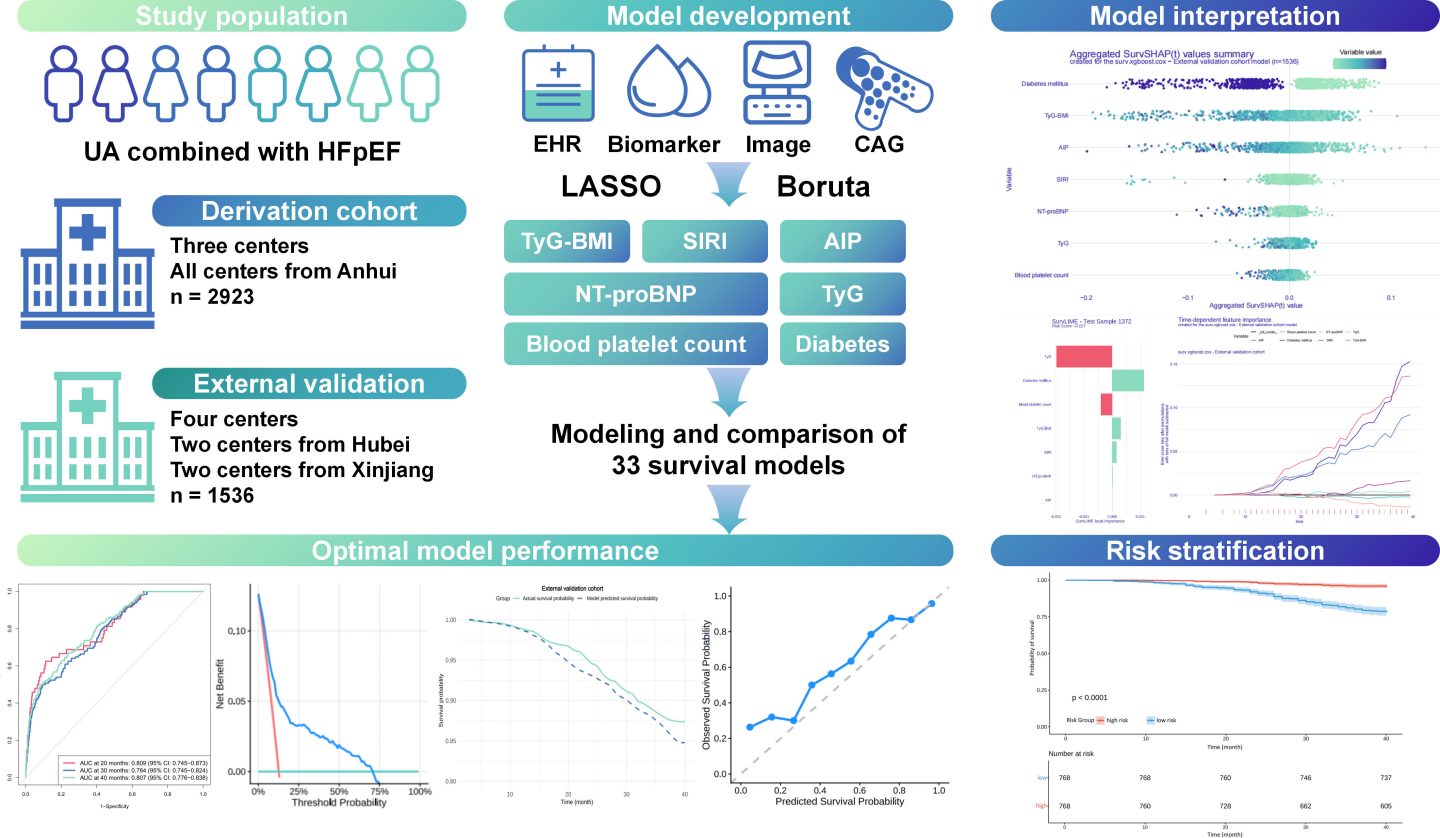
Visual summary of the predictive framework for patients with unstable angina (UA) and heart failure with preserved ejection fraction (HFpEF). AIP: atherogenic index of plasma; CAG: coronary angiography; EHR: electronic health record; LASSO: least absolute shrinkage and selection operator; NT-proBNP: N-terminal pro-brain natriuretic peptide; SIRI: systemic inflammation response index; TyG: triglyceride-glucose.

This multicenter cohort study represents the first comprehensive attempt to develop and validate ML-based predictive models for MACEs in the clinically challenging dual phenotype of HFpEF and UA. Our findings demonstrate that a surv.xgboost.cox algorithm incorporating 7 critical predictors can effectively stratify risk in this complex patient population with satisfactory discriminative ability, calibration, and clinical utility. Moreover, the translation of our prediction model into a publicly accessible web−based calculator facilitates its integration into clinical workflows and decision-making processes (Figure 9).

### Comparison With Previous Research

Previous studies focused on developing risk assessment models tailored for HFpEF patients to aid clinicians in better understanding and managing the disease for optimal outcomes [28-32]. Despite these advancements, it is important to emphasize that HFpEF is a highly heterogeneous syndrome with phenotypes complicated by comorbidities, including obesity, diabetes, hypertension, and coronary artery disease [1,2]. These comorbidities interact synergistically, forming a distinct pathophysiological network with varying prognostic effects across patient populations [3,4]. Patients often present with comorbidities as their first point of contact in the clinical setting. However, clinicians frequently fail to identify those with early-stage HFpEF due to the lack of overt symptoms, causing delays in the initiation of appropriate treatment. Consequently, it is imperative to develop refined risk stratification strategies tailored to the distinct clinical phenotypes of HFpEF. Notably, UA is common in the HFpEF patient population and correlates with an increased incidence of MACCEs in this population [2].

Efforts have recently begun to address the need to manage the HFpEF comorbid population. Wang et al [33] developed a nomogram model for HFpEF patients with ischemic cardiomyopathy after coronary artery bypass grafting, integrating diabetes, hypertension, SII, and NT-proBNP. Additionally, they developed an ML-based model for predicting adverse outcomes in patients with symptomatic aortic stenosis and HFpEF after transcatheter aortic valve replacement using predictors, including age, NT-proBNP, fasting blood glucose, triglyceride or HDL ratio, and apolipoprotein B [34]. Iwakura et al [35] applied the WATCH-DM risk score to estimate clinical outcomes in patients with HFpEF and diabetes, demonstrating its capability to identify individuals at an elevated risk of mortality. However, these models’ relatively modest performance metrics highlight the challenges of generalizing traditional prediction tools to specific subpopulations. An integrated approach that combines clinical features, imaging data, and multiple biomarker panels is essential for accurate risk stratification and individualized treatment strategies, thereby enhancing the applicability of traditional predictive tools across diverse HFpEF populations.

Our study is the first to specifically target the high-risk and understudied population of patients with UA and HFpEF. Our study enrolled a large population from 3 distinct regions in China and utilized a comprehensive multicenter dataset with multimodal data, including clinical texts, biomarkers, and imaging data. By leveraging ML techniques to integrate these diverse data types, we developed a MACE prediction model within a multicenter cohort. Compared to previous studies [30,35], our model integrates traditional risk factors (diabetes mellitus) and metabolic and inflammatory biomarkers (TyG, AIP, TyG-BMI, and SIRI). This integration allows for more sensitive detection of ischemia-induced pathological processes. We employed 33 ML algorithms to individually fit each of the 7 predictive variables to determine the optimal performance. The optimal surv.xgboost.cox model performed well in external validation cohorts and has been implemented in clinical practice through an online calculator, providing an accurate tool for the detection of high-risk UA-HFpEF patients. Additionally, our model provides clinicians with a valuable tool for identifying patients with HFpEF and UA, especially in routine health examinations or primary medical facilities with limited experience in managing these conditions. Moreover, the explainable method indicates that the contributions of the included features are transparent and highly consistent with the importance estimated by previous studies [36,37]. The identification of key comorbidity predictors and their interaction effects offers novel insights into the complex pathophysiology underlying this condition. Integrating this model into clinical practice could improve risk stratification and inform personalized management strategies for this high-risk population.

The incorporation of multiple indicators in our study highlights the pivotal role of inflammation and metabolic dysregulation in the pathophysiology of UA concurrent with HFpEF. Diabetes mellitus, a prevalent comorbidity in HFpEF, continues to be a crucial determinant of disease progression and adverse outcomes, aligning with previous research that underscores its significant impact on both HFpEF and UA [38]. Both TyG and TyG-BMI are reliable surrogate markers for insulin resistance and have been associated with various cardiovascular events, including acute coronary syndrome, HF, and stroke, demonstrating independent prognostic value in both HFpEF and UA [39-41]. Similarly, the AIP serves as an independent feature of insulin resistance and cardiometabolic risk, with established efficacy in predicting arterial stiffness and HF [39-41]. Systemic inflammation represents a pivotal pathophysiological mechanism in the complex etiology and progression of HFpEF [42,43]. Our findings indicate that inflammatory biomarkers, particularly the SIRI, suggest that inflammation is not merely an epiphenomenon but rather a central mediator in the pathogenesis of HFpEF, especially in the presence of coronary instability. When integrated into risk prediction models, these metabolic and inflammatory biomarkers collectively improve risk stratification and optimize the management of patients with UA and comorbid HFpEF. This pragmatic selection of variables enables implementation across diverse health care settings without requiring specialized equipment or expertise, addressing a key barrier to the clinical adoption of prediction models. Furthermore, the clinical accessibility and cost-effectiveness of these biomarkers promote their widespread application in primary care settings, underscoring their potential for large-scale risk assessment and patient management. Our web-based calculator simplifies complex mathematical modeling into an intuitive interface that can be seamlessly integrated into personalized prevention decision-making during time-constrained clinical encounters, representing a significant advancement over traditional risk scores that often require manual calculation or memorization of complex algorithms.

### Future Work

It is noteworthy that with the advancement of large language models (LLMs), their potential in disease management, decision support, and risk prediction, particularly in cardiovascular diseases, is becoming increasingly evident [44-47]. Particularly, the ability of LLMs to engage in natural language interactions with both doctors and patients could offer more intuitive explanations for our model’s predictions in the future [48]. Our next steps involve integrating LLMs with our multimodal data−driven prognostic model, which would further enhance the model’s interpretability and provide clinicians with more personalized and comprehensible risk assessments. This direction holds the potential to significantly advance precision medicine and decision support systems in cardiovascular disease management.

### Study Limitations

This study has several limitations. First, the research was conducted in China with participants primarily recruited from local populations, and the treatment approaches were specifically tailored to the observed clinical scenarios. As a result, applying these findings to a global context may introduce potential biases, a common limitation in similar predictive models. Second, despite our rigorous feature selection process, unmeasured confounders and residual biases cannot be excluded. This underscores the need for prospective validation to confirm the clinical applicability and reproducibility of our risk stratification tool. Third, due to data-sharing restrictions and the costs associated with data annotation, patient recruitment was limited to 7 major hospitals in China, which may limit the broader applicability of the model. Fourth, while the 7 predictive variables selected by the development model are present on admission, intraoperative factors and lifestyle choices significantly influence the risk of MACEs in real-world clinical settings. Fifth, we acknowledge that there were baseline imbalances in certain key variables, such as D-dimer levels, between the derivation and validation cohorts. While efforts were made to address some of these imbalances by incorporating data from an additional center, the persistence of these differences remains a limitation that should be considered when interpreting the results. Sixth, the findings may be subject to biases such as recall bias or data limitations inherent in the electronic health record system. These potential biases highlight the need for further validation in diverse cohorts. Additionally, the model’s performance showed a relatively high C-index of 0.954 in the derivation cohort but a drop to 0.788 in the external validation cohort. While 0.788 is a respectable validation score, this difference suggests that the model may have overfitted to the training data. This overfitting could be due to the optimization of hyperparameters for the training set, and the high performance in the derivation cohort likely reflects some memorization of the training data. Despite this, the model generalizes well enough for clinical use, as reflected by its external validation score. Finally, despite including 72 candidate variables, our model does not capture several important confounders: longitudinal medication adherence and dose optimization, socioeconomic factors (education, income, insurance), lifestyle behaviors (physical activity, diet, psychosocial factors), temporal variations from evolving therapies, and genetic variants or emerging biomarkers. Our multicenter design, regularized machine learning approach, and external validation mitigate many of these limitations, but future prospective studies with comprehensive data collection are needed to address residual confounding and further validate the model’s applicability.

### Conclusions

This study developed and externally validated a survival-based ML model incorporating 7 clinical variables to predict MACEs in patients with HFpEF and UA. The findings suggest that integrating metabolic and inflammatory biomarkers with cardiac function parameters may improve risk stratification in these patients. Prospective validation studies are warranted to evaluate the model’s impact on clinical decision-making and patient outcomes.

## Supplementary material

10.2196/78402 Multimedia Appendix 1 Study criteria, feature selection outputs, model parameters and performance metrics, interpretability analyses, and individualized major adverse cardiovascular event risk predictions.

## Abbreviations

AIP: atherogenic index of plasma
AUC: area under the curve
C-index: concordance index
C/D AUC: cumulative/dynamic area under the curve
HDL-C: high-density lipoprotein cholesterol
HF: heart failure
HFpEF: heart failure with preserved ejection fraction
HFrEF: heart failure with reduced ejection fraction
LASSO: least absolute shrinkage and selection operator
LLM: large language model
MACE: major adverse cardiovascular event
ML: machine learning
NLR: neutrophil-to-lymphocyte ratio
NPV: negative predictive value
NT-proBNP: N-terminal pro-brain natriuretic peptide
PDP: partial dependence survival profile
PLR: platelet-to-lymphocyte ratio
PPV: positive predictive value
ROC: receiver operating characteristic
SII: systemic immune-inflammation index
SIRI: systemic inflammation response index
SurvSHAP(t): Shapley Additive Explanations for survival analysis
TyG: triglyceride-glucose
UA: unstable angina
