# Supplementary material for: Multimodal Data–Driven Explainable Prognostic Model for Major Adverse Cardiovascular Events Prediction in Patients With Unstable Angina and Heart Failure With Preserved Ejection Fraction: Multicenter, Cross-Regional Cohort Study
Source: J Med Internet Res. 2025 Dec 15;27:e78402. doi: 10.2196/78402 (PMC12705130; doi:10.2196/78402)

Table S1. Inclusion and exclusion criteria.

**Inclusion criteria**
Age between 18 and 95 years
Heart failure with preserved ejection fraction
Unstable angina
Successful coronary angiography
Follow-up was completed.
**Exclusion criteria**
Acute myocardial infarction
Implantation of pacemaker
Acute- and chronic-phase inflammatory responses
Autoimmune diseases
Left ventricular ejection fraction <50%
Malignant tumor
Severe valvular heart disease
Dilated cardiomyopathy
Rheumatic heart disease

Cor pulmonale

Myocarditis or cardiomyopathy

Infectious or severe liver or kidney disease

Lacked data on fasting triglyceride (TG) and fasting blood-glucose (FBG)

Patients without the results of cardiac uhrasonography

Missing data for any variable

Refusal to participate in follow-up.

Table S2. The Variance Inflation Factor (VIF) of Seven Feature.

Feature	VIF	Multicollinearity test (VIF >= 5)
Diabetes mellitus	1.149	No
Blood platelet count	1.021	No
SIRI	1.026	No
NT-proBNP	1.017	No
TyG	3.050	No
TyG-BMI	1.265	No
AIP	2.948	No

Table S3. More Performance of surv.xgboost.cox model.

Dataset	Time_Point	Accuracy	Sensitivity/Recall	Specificity	PPV	NPV	F1_Score	AUC	AUC_CI
derivation cohort	20-month	0.96	0.335	0.996	0.844	0.963	0.48	0.96	(0.951-0.970)
derivation cohort	30-month	0.937	0.435	0.991	0.838	0.942	0.573	0.972	(0.966-0.978)
derivation cohort	40-month	0.933	0.565	0.994	0.936	0.932	0.705	0.98	(0.975-0.985)
validation cohort	20-month	0.964	0.2	0.99	0.4	0.974	0.267	0.809	(0.745-0.873)
validation cohort	30-month	0.911	0.257	0.975	0.5	0.931	0.34	0.784	(0.745-0.824)
validation cohort	40-month	0.893	0.34	0.972	0.641	0.911	0.444	0.807	(0.776-0.838)

Table S4. Optimal parameters of 33 machine learning models in predicting MACEs rates.

Model	Optimal parameter
surv.gbm	distribution = coxph; keep.data = FALSE; n.cores = 1; n.trees = 350; interaction.depth = 6; shrinkage = 0.01; n.minobsinnode = 5; bag.fraction = 0.7
surv.xgboost.cox	nrounds = 525; nthread = 1; verbose = 0; max_depth = 9; eta = 0.01; gamma = 5; subsample = 0.7; colsample_bytree = 1; min_child_weight = 1
surv.rpart	xval = 0; minsplit = 50; minbucket = 5; cp = 0.001; maxdepth = 5
surv.cforest	cores = 1; teststat = quadratic; testtype = Univariate; mincriterion = 0.9; saveinfo = FALSE; ntree = 500; mtry = 7
surv.xgboost.aft	nrounds = 525; nthread = 1; verbose = 0; max_depth = 9; eta = 0.01; gamma = 5; subsample = 0.7; colsample_bytree = 1; min_child_weight = 1
surv.blackboost	family = coxph; mstop = 100; nu = 0.3775
surv.ranger	num.threads = 1; num.trees = 1050; mtry = 3; min.node.size = 12; sample.fraction = 0.75
surv.gamboost	family = coxph; baselearner = bols; mstop = 100; nu = 0.3
surv.glmboost	family = coxph; mstop = 100; nu = 0.3
surv.mboost	family = coxph; baselearner = bols; mstop = 100; nu = 0.3
surv.cv_glmnet	use_pred_offset = TRUE; alpha = 1; nfolds = 10

surv.cv_coxboost	Default parameters
surv.aorsf	n_thread = 0; n_tree = 325; mtry = 7; leaf_min_obs = 15; split_min_obs = 16
surv.bart	mc.cores = 1; quiet = TRUE; importance = count; which.curve = 0.5; ntree = 62; ndpost = 163; nskip = 200
surv.coxboost	stepno = 87; penalty = 10; stepsize.factor = 0.5
surv.ctree	mincriterion = 0.9225; minsplit = 50; minbucket = 20
surv.parametric	form = aft; dist = exponential
surv.glmnet	use_pred_offset = TRUE; alpha = 0; s = 0.001
surv.coxph	Default parameters
surv.penalized	trace = FALSE; lambda1 = 0; lambda2 = 0
surv.flexible	Default parameters
surv.priority_lasso	blocks = list(1:5, 6:7); max.coef = c(3, 5); block1.penalization = FALSE; lambda.type = lambda.1se; alpha = 0.1
surv.loghaz	Default parameters
surv.rfsrc	ntime = 0; ntree = 1050; mtry = 4; nodesize = 3; sampsize = 200
surv.coxtime	epochs = 9; batch_size = 12; dropout = 0.15; frac = 0.375
surv.deepsurv	Default parameters
surv.akritas	Default parameters
surv.deephit	batch_norm = FALSE; epochs = 20; batch_size = 44; dropout = 0.225; frac = 0.45

surv.dnnsurv	optimizer = adam; verbose = 0; epochs = 18; batch_size = 40; cuts = 10; lr = 0.0105
surv.kaplan	Default parameters
surv.nelson	Default parameters
surv.svm	type = regression; gamma = 0.0325; mu = 2; kernel = rbf_kernel
surv.pchazard	batch_norm = FALSE; epochs = 15; batch_size = 48; dropout = 0.175; frac = 0.8

Figure S1. Dual-Method Feature Selection via LASSO Regression and Boruta Algorithm for Key Clinical Predictors. (A) Optimal λ selection in LASSO using 10-fold cross-validatio. (B) Coefficient trajectories of 72 candidate variables across λ values. (C) Boruta's feature importance dynamics over 100 iterations: confirmed (green), tentative (yellow), and shadow features (red). (D) Evolution of Z-scores for variables selected by Boruta.

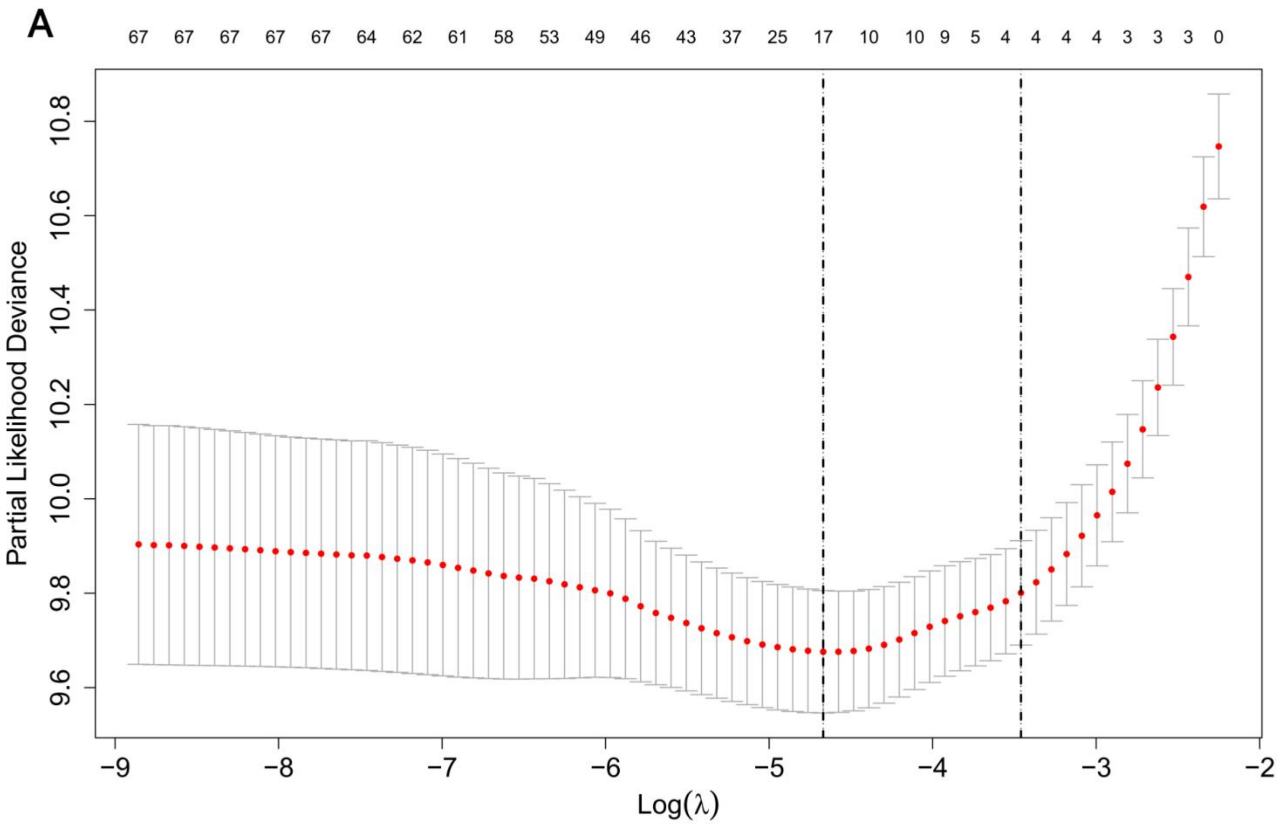

B

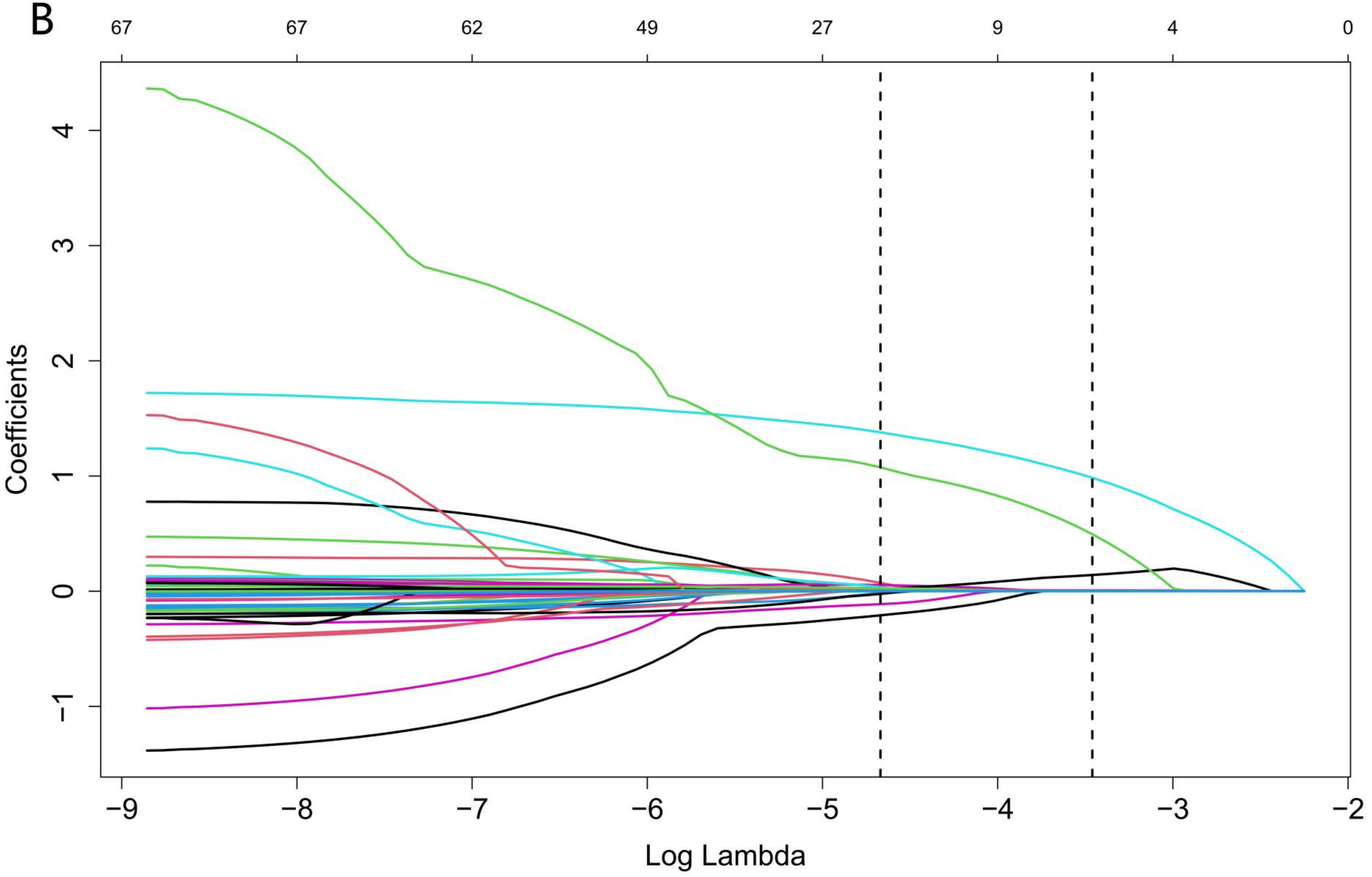

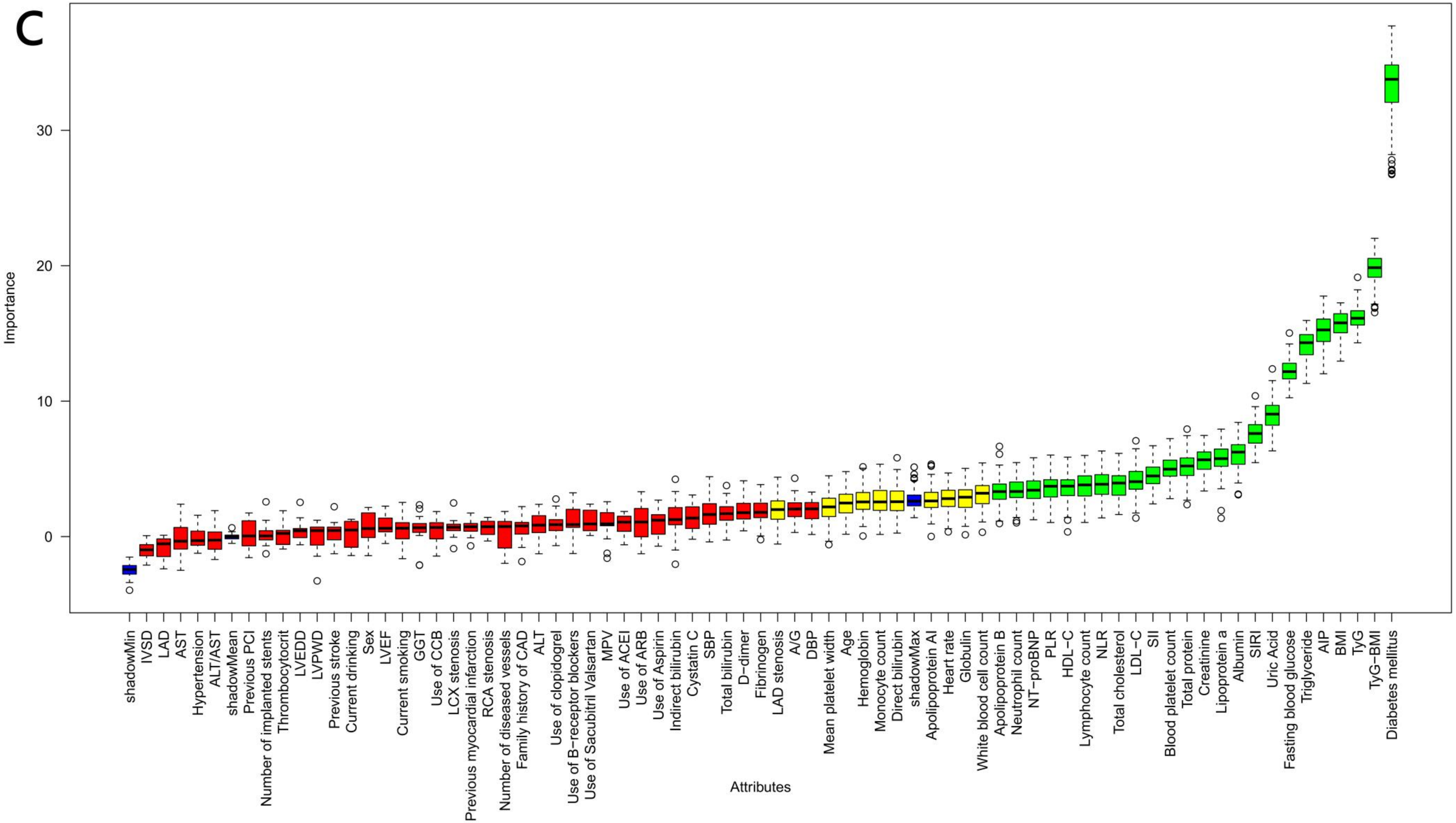
C
Importance
Attributes
0
10
20
30
shadowMin
IVSD
LAD
AST
Hypertension
ALT/AST
shadowMean
Previous PCI
Number of implanted stents
Thrombocytocrit
LVEDD
LVPWD
Previous stroke
Current drinking
Sex
LVEF
Current smoking
GGT
Use of CCB
LCX stenosis
Previous myocardial infarction
RCA stenosis
Number of diseased vessels
Family history of CAD
ALT
Use of clopidogrel
Use of B−receptor blockers
Use of Sacubitril Valsartan
MPV
Use of ACEI
Use of ARB
Use of Aspirin
Indirect bilirubin
Cystatin C
SBP
Total bilirubin
D−dimer
Fibrinogen
LAD stenosis
A/G
DBP
Mean platelet width
Age
Hemoglobin
Monocyte count
Direct bilirubin
shadowMax
Apolipoprotein AI
Heart rate
Globulin
White blood cell count
Apolipoprotein B
Neutrophil count
NT−proBNP
PLR
HDL−C
Lymphocyte count
NLR
Total cholesterol
LDL−C
SII
Blood platelet count
Total protein
Creatinine
Lipoprotein a
Albumin
SIRI
Uric Acid
Fasting blood glucose
Triglyceride
AIP
BMI
TyG
TyG−BMI
Diabetes mellitus

D

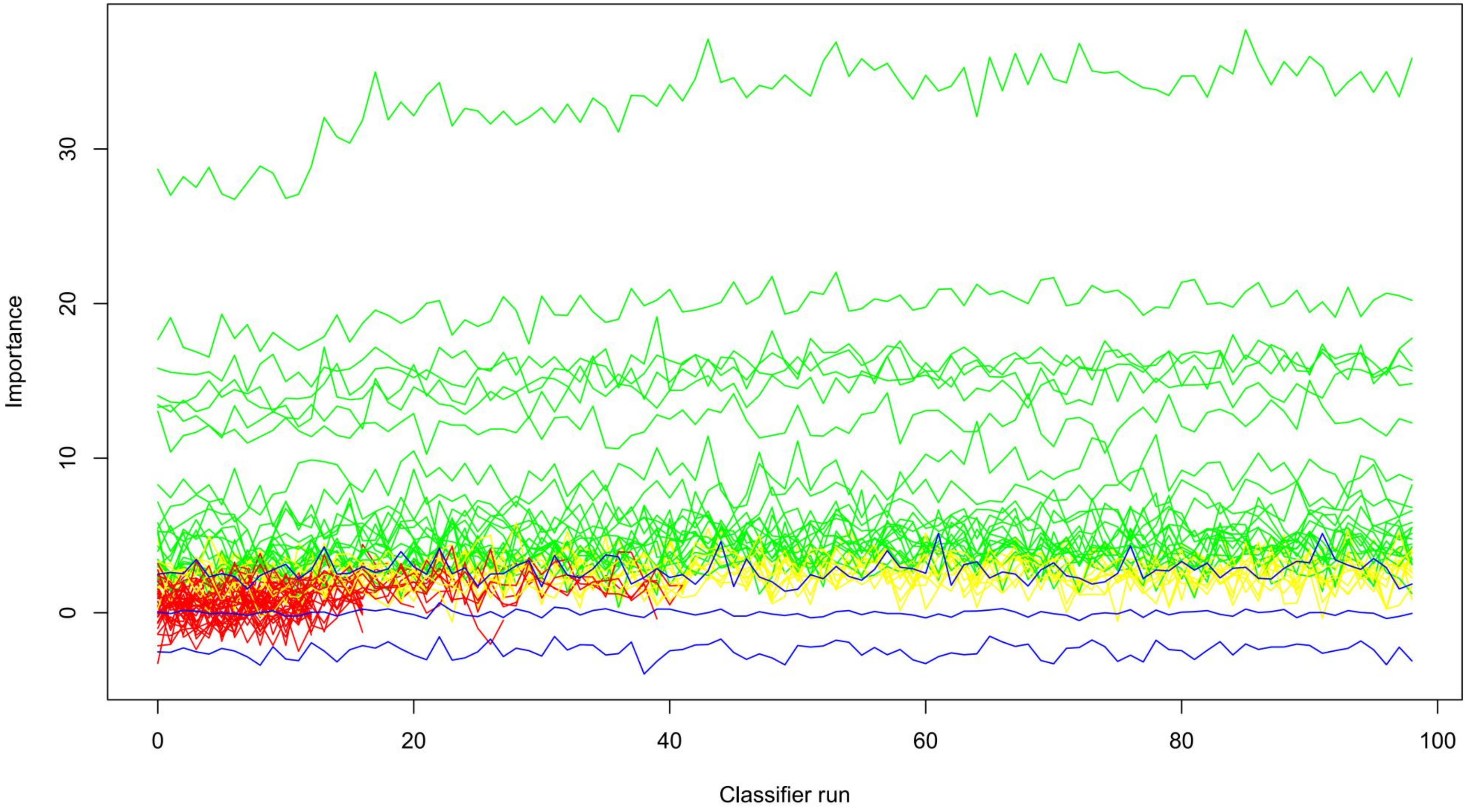

Figure S2.Heatmap of the distribution of seven key patient features across the cohort.

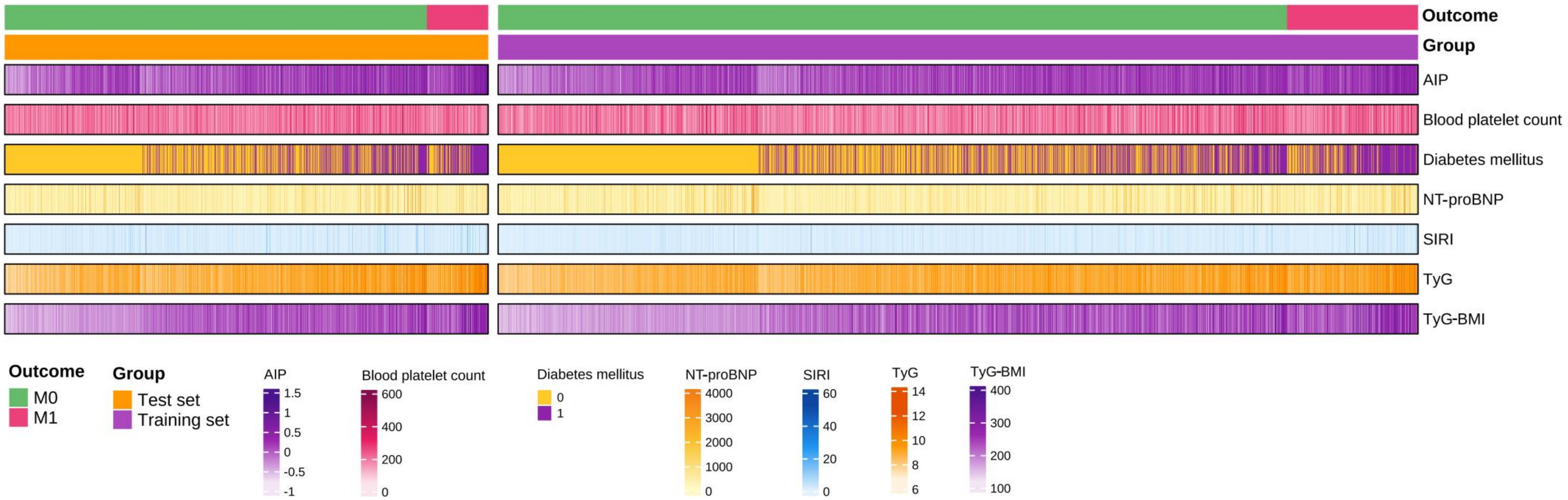

Figure S3. Overall performance metrics of the surv.xgboost.cox model based on the Survex interpreter in the derivation cohort. (A) Time-dependent Brier score and C/D AUC metrics, (B) Overall C-index, Brier score, and C/D AUC metrics.

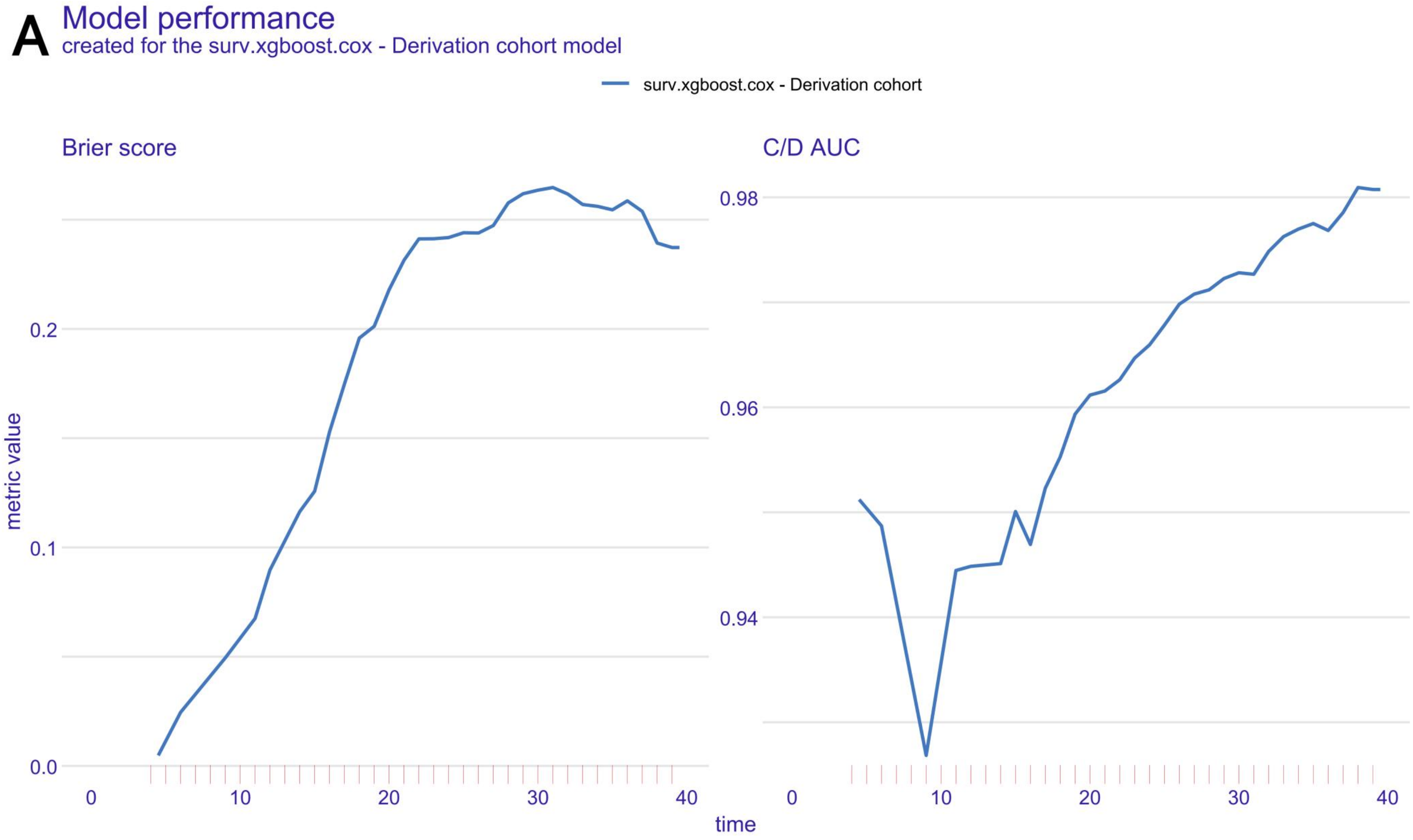

B

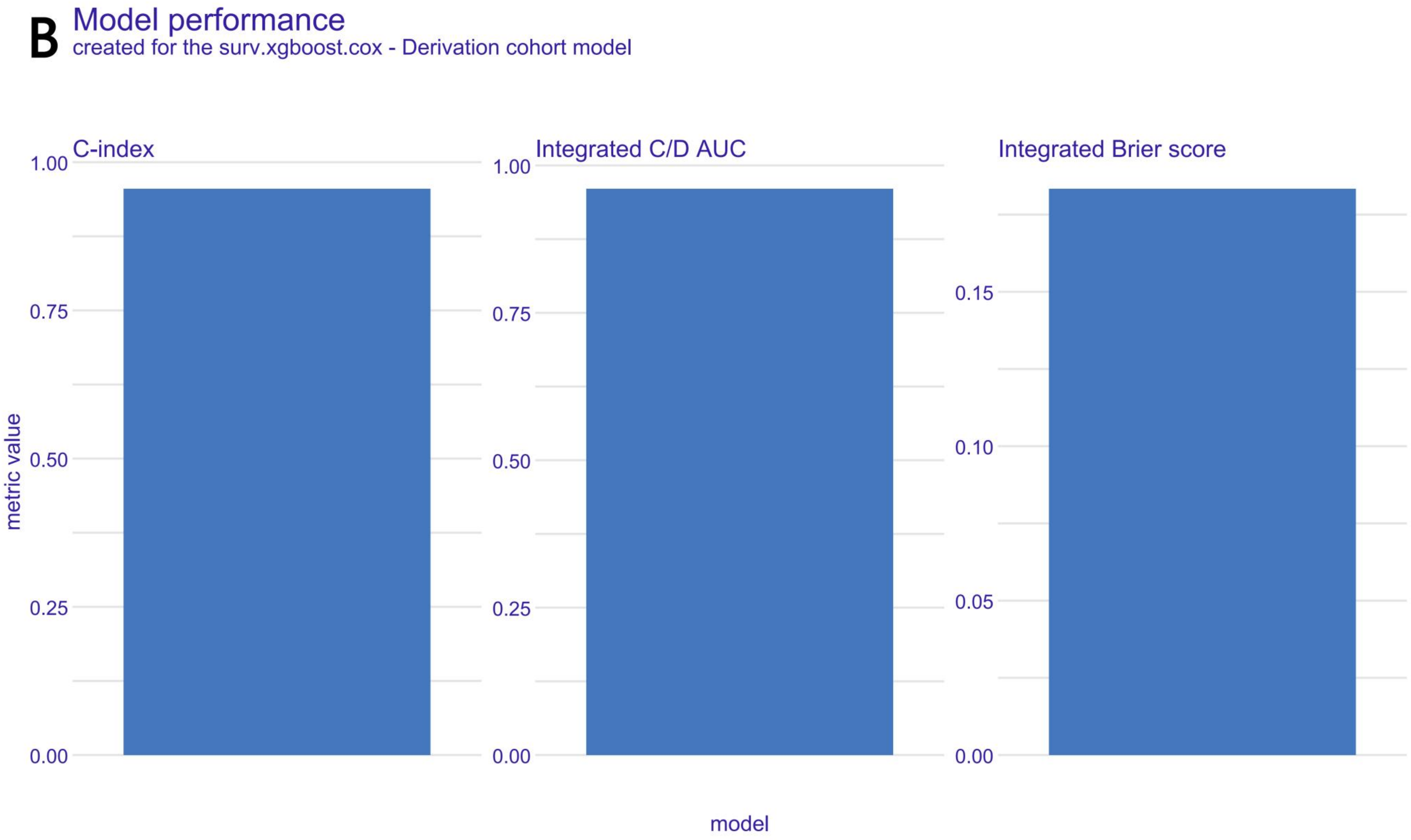
Model performance
created for the surv.xgboost.cox - Derivation cohort model
C-index
Integrated C/D AUC
Integrated Brier score
metric value
model
1.00
0.75
0.50
0.25
0.00
0.15
0.10
0.05

Figure S4. Decision curve analysis (DCA) curves at 20, 30, and 40 months for the derivation and external validation cohorts.

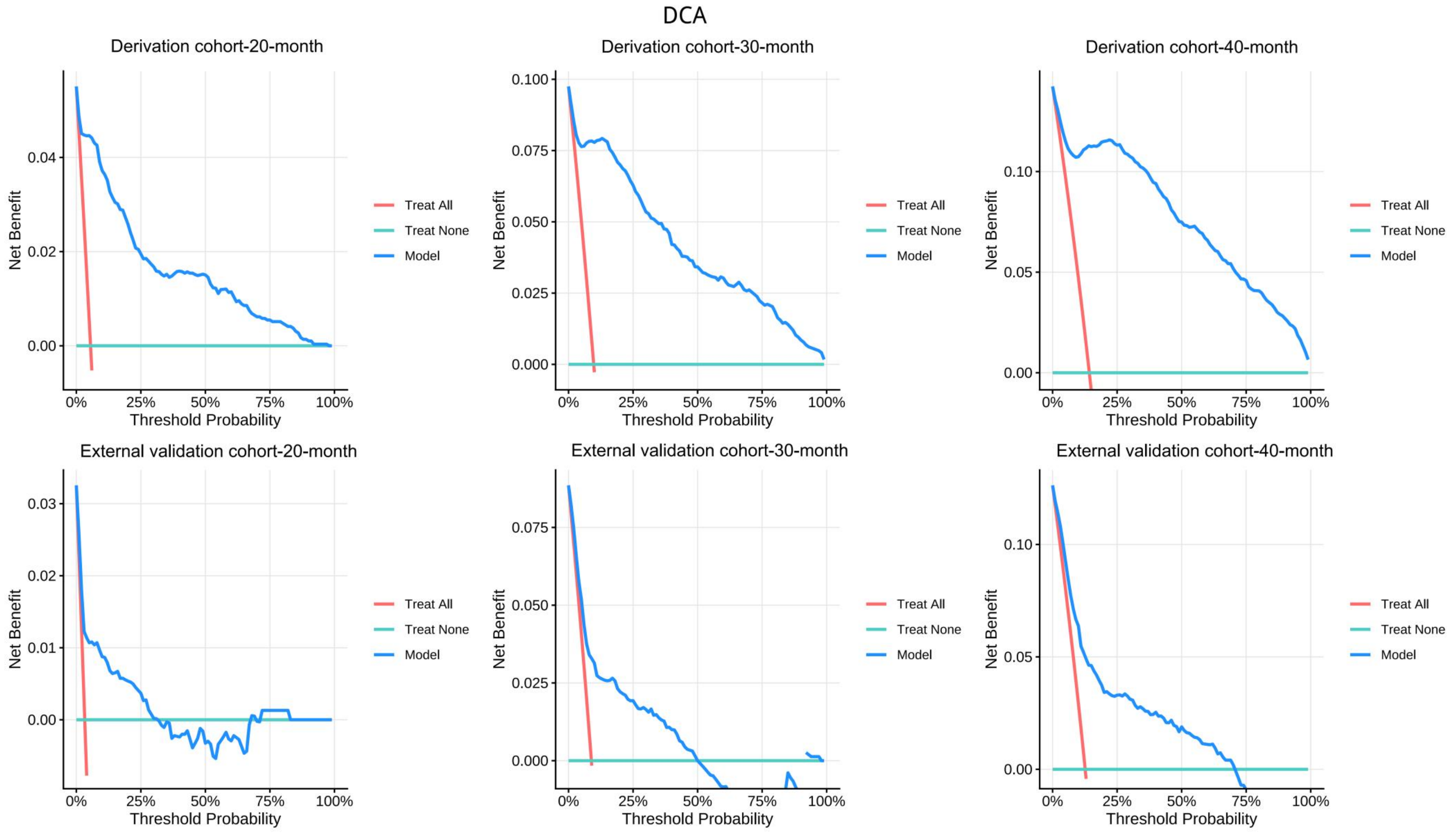

Figure S5. Variable importance ranking based on C/D AUC with time-dependent line plot for feature significance.

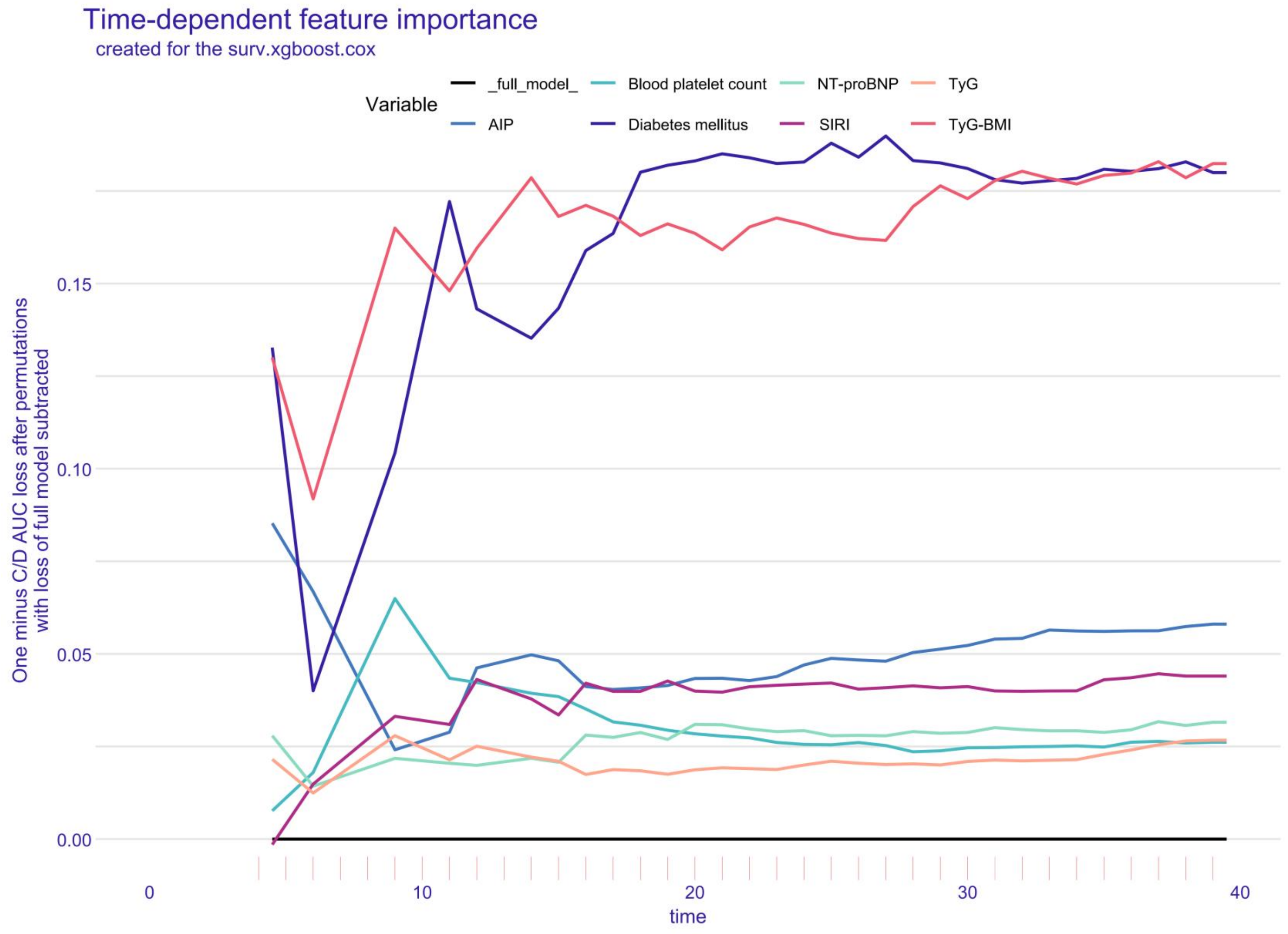

Figure S6. Partial dependence survival profiles of the marginal effects of feature variables on the final survival outcome.

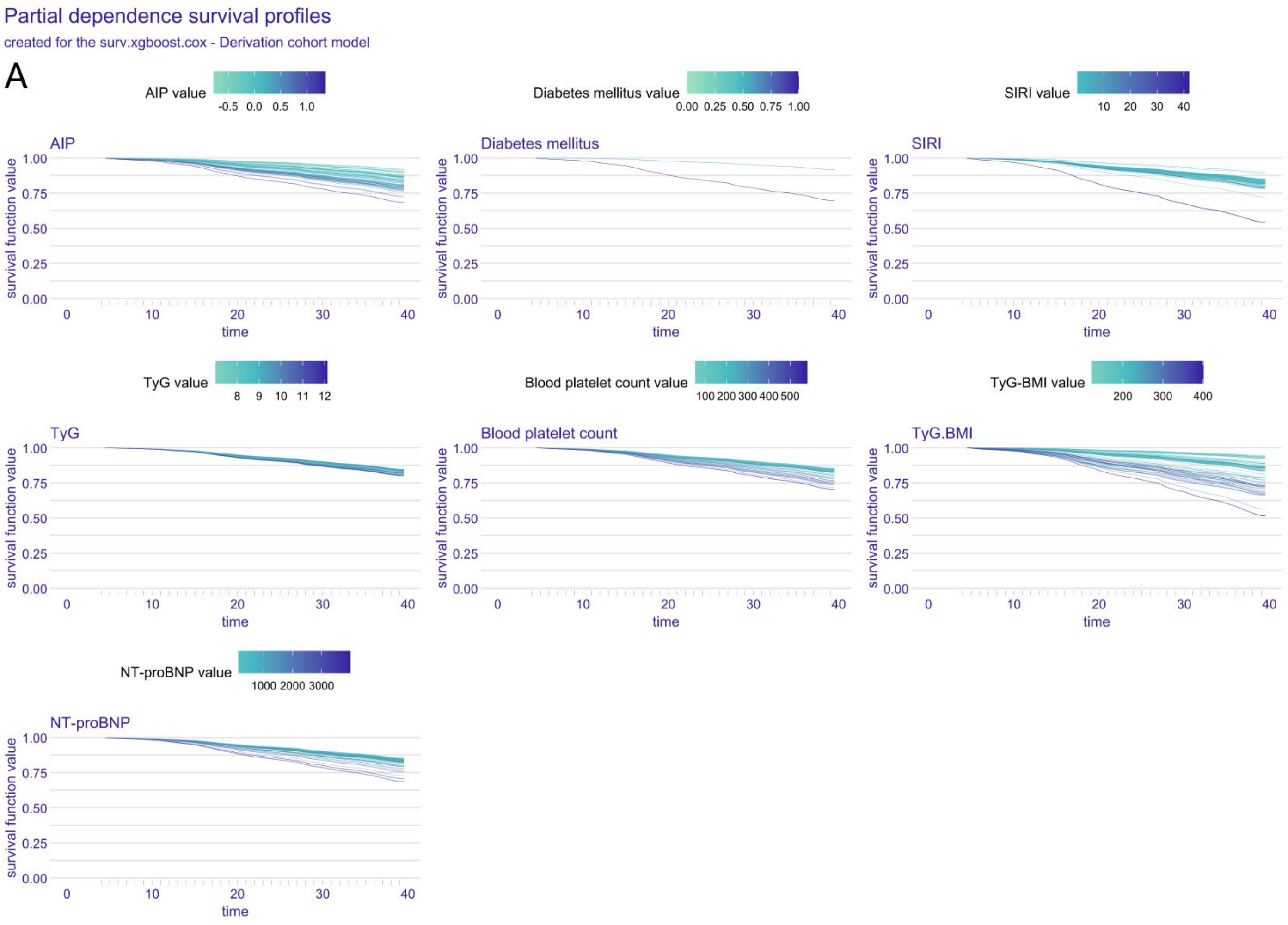

Figure S7. SurvSHAP(t)-Based Marginal Effects Analysis of Key Predictive Features. Univariate dependence plots showing the time-dependent linear relationships between seven key features (diabetes mellitus, blood platelet count, NT-proBNP, AIP, SIRI, TyG, and TyG-BMI) and the model-predicted 40-month MACEs risk.

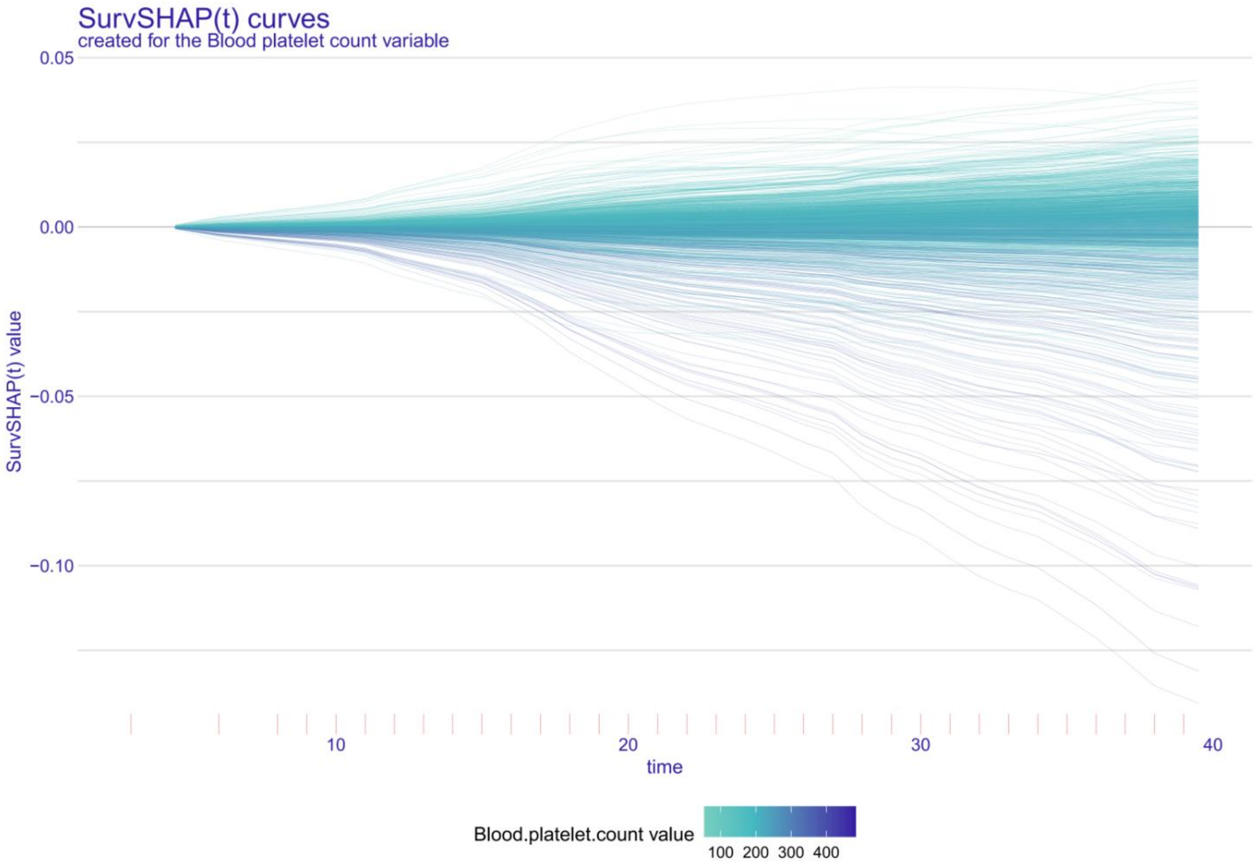

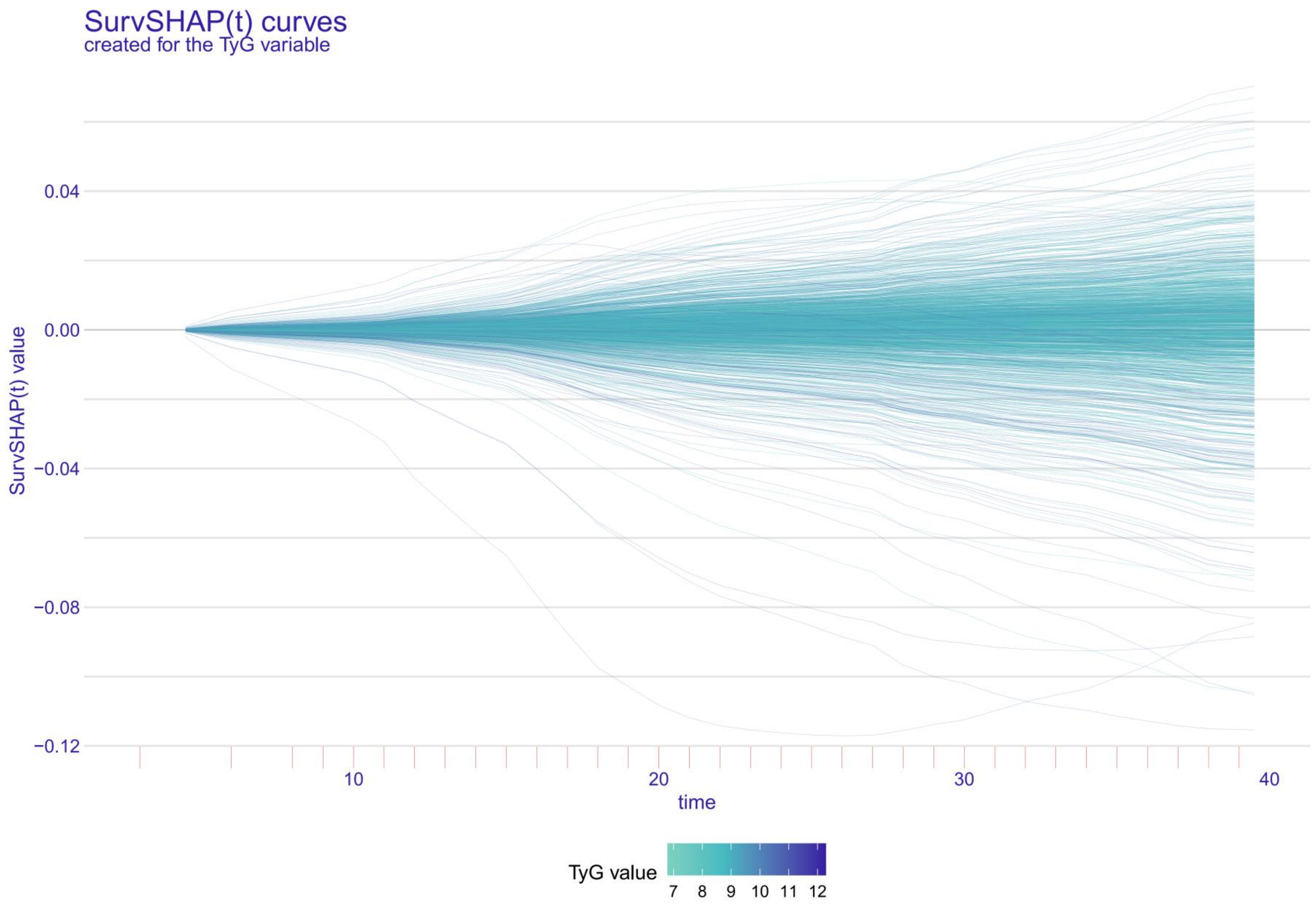
SurvSHAP(t) curves
created for the TyG variable
SurvSHAP(t) value
0.04
0.00
−0.04
−0.08
−0.12
10
20
30
40
time
TyG value
7 8 9 10 11 12

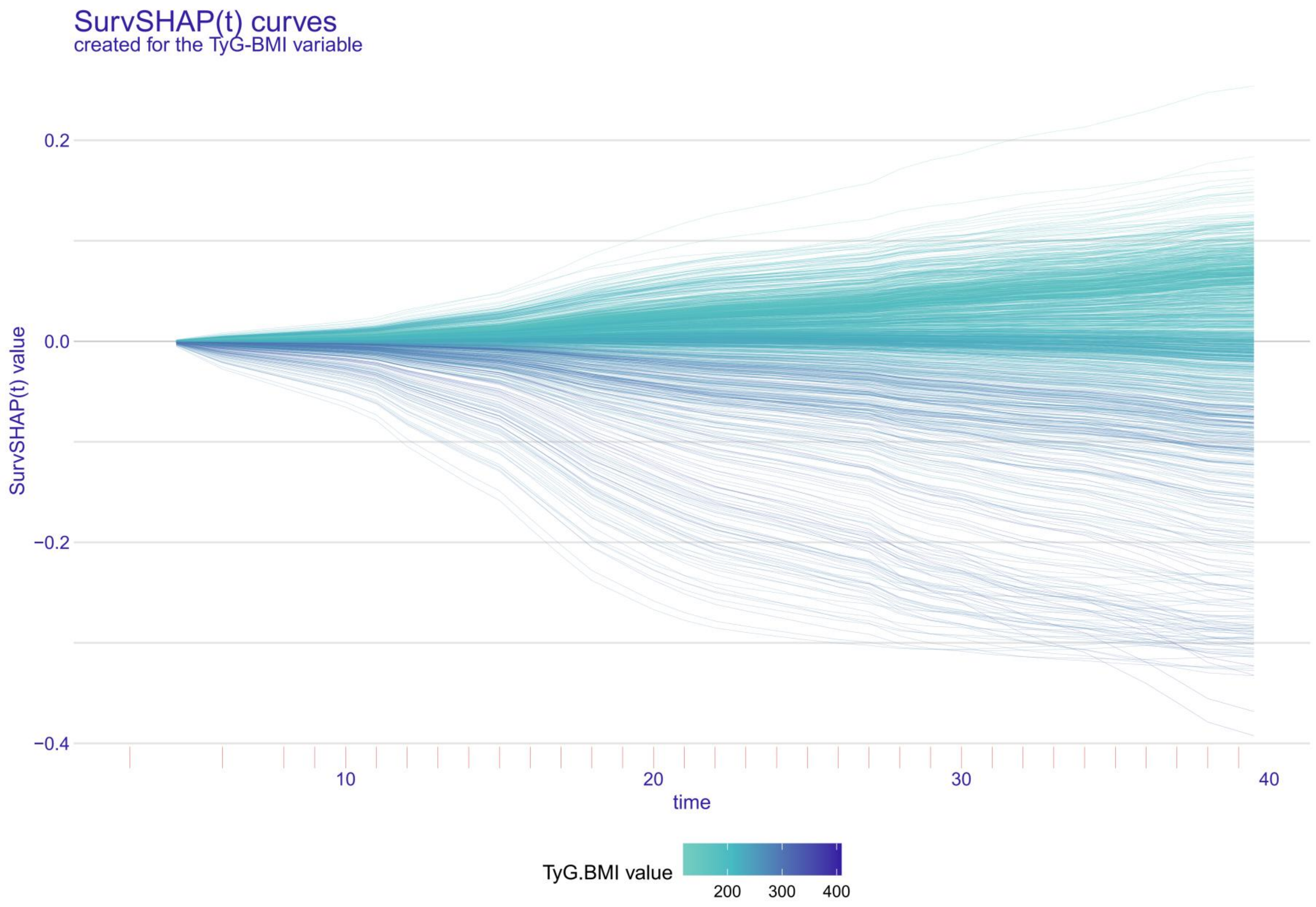
SurvSHAP(t) curves
created for the TyG-BMI variable
0.2
0.0
−0.2
−0.4
SurvSHAP(t) value
10
20
30
40
time
TyG.BMI value
200
300
400

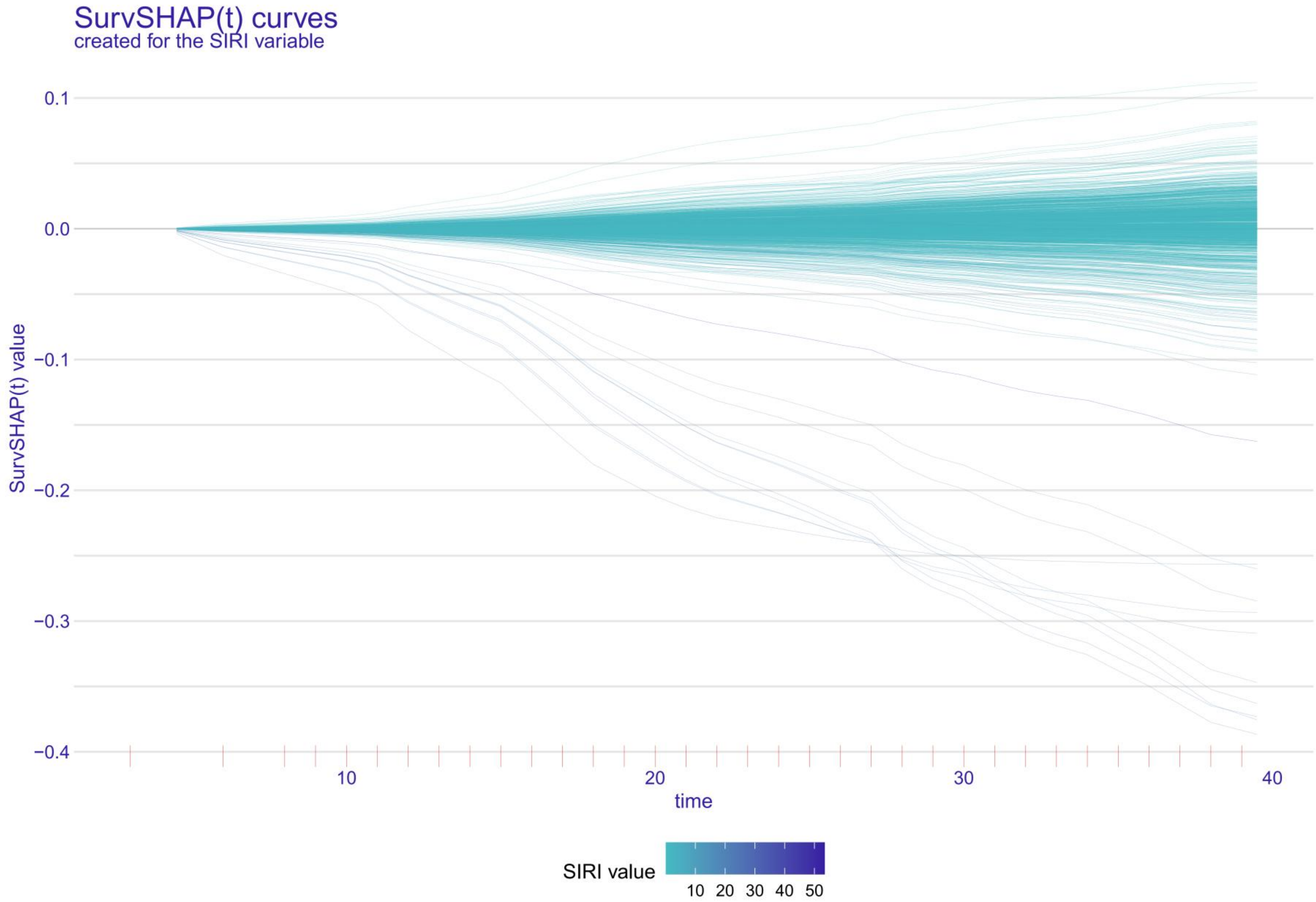
SurvSHAP(t) curves
created for the SIRI variable
0.1
0.0
−0.1
−0.2
−0.3
−0.4
SurvSHAP(t) value
10
20
30
40
time
SIRI value
10 20 30 40 50

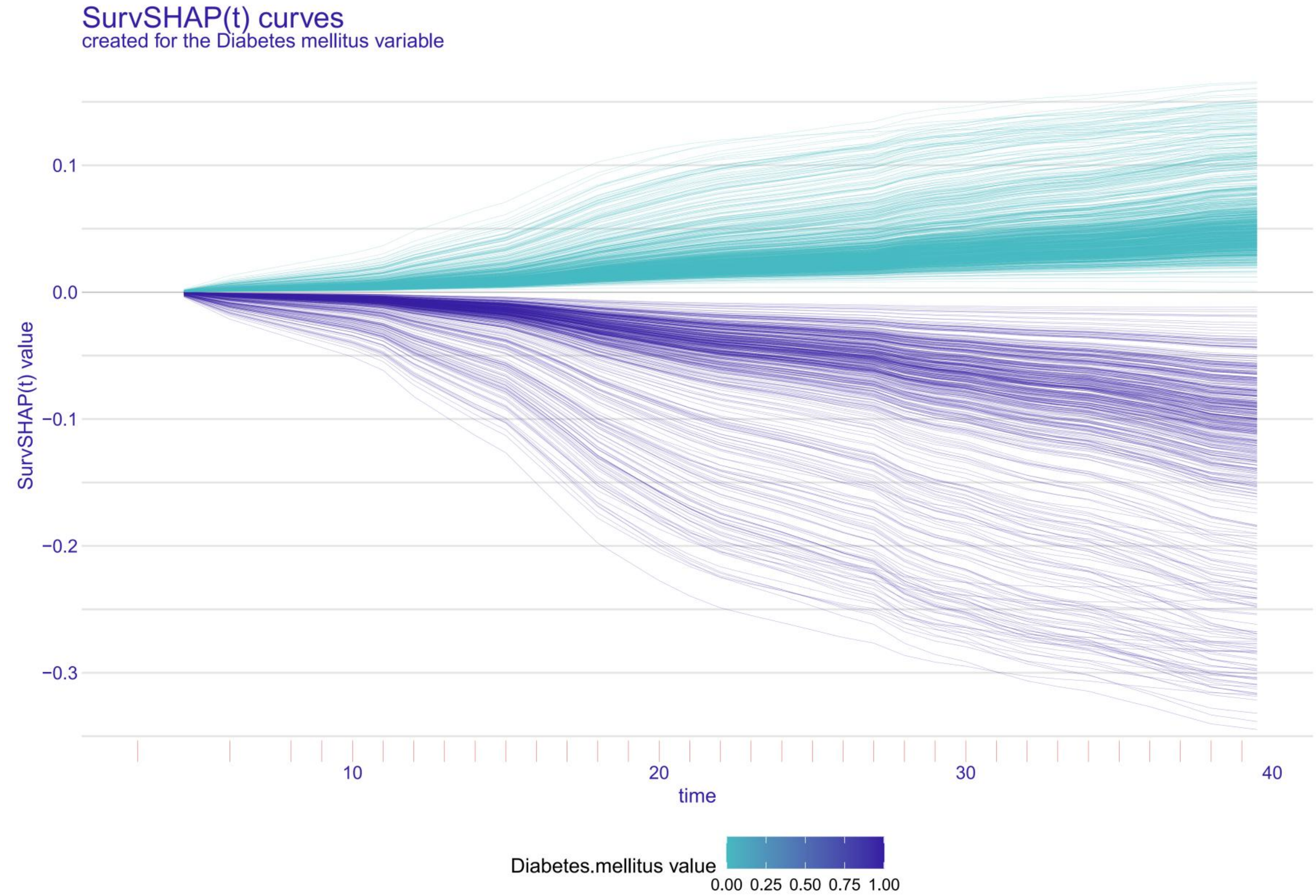
SurvSHAP(t) curves
created for the Diabetes mellitus variable
SurvSHAP(t) value
0.1
0.0
−0.1
−0.2
−0.3
10
20
30
40
time
Diabetes.mellitus value
0.00 0.25 0.50 0.75 1.00

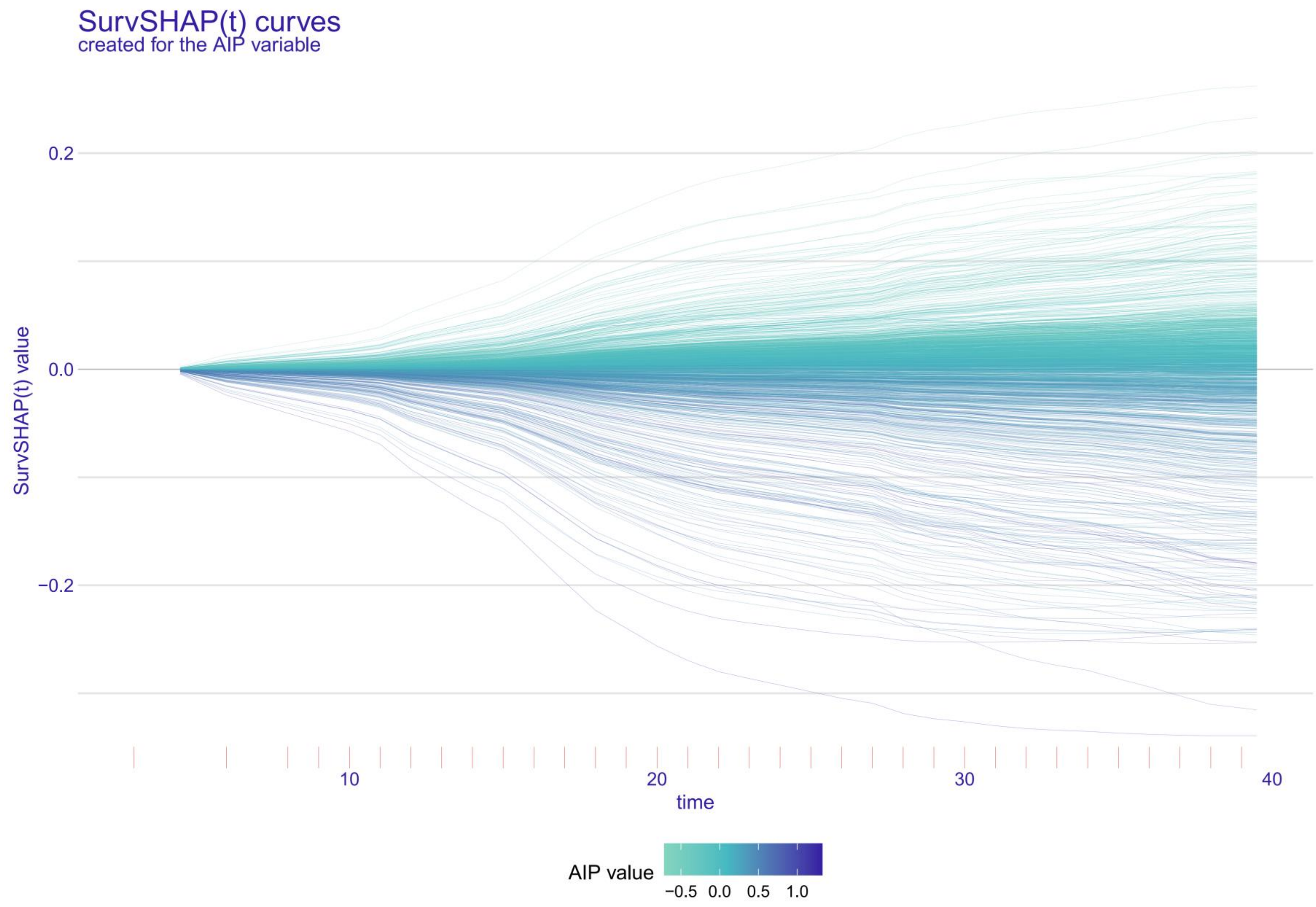
SurvSHAP(t) curves
created for the AIP variable
0.2
0.0
−0.2
SurvSHAP(t) value
10
20
30
40
time
AIP value
−0.5
0.0
0.5
1.0

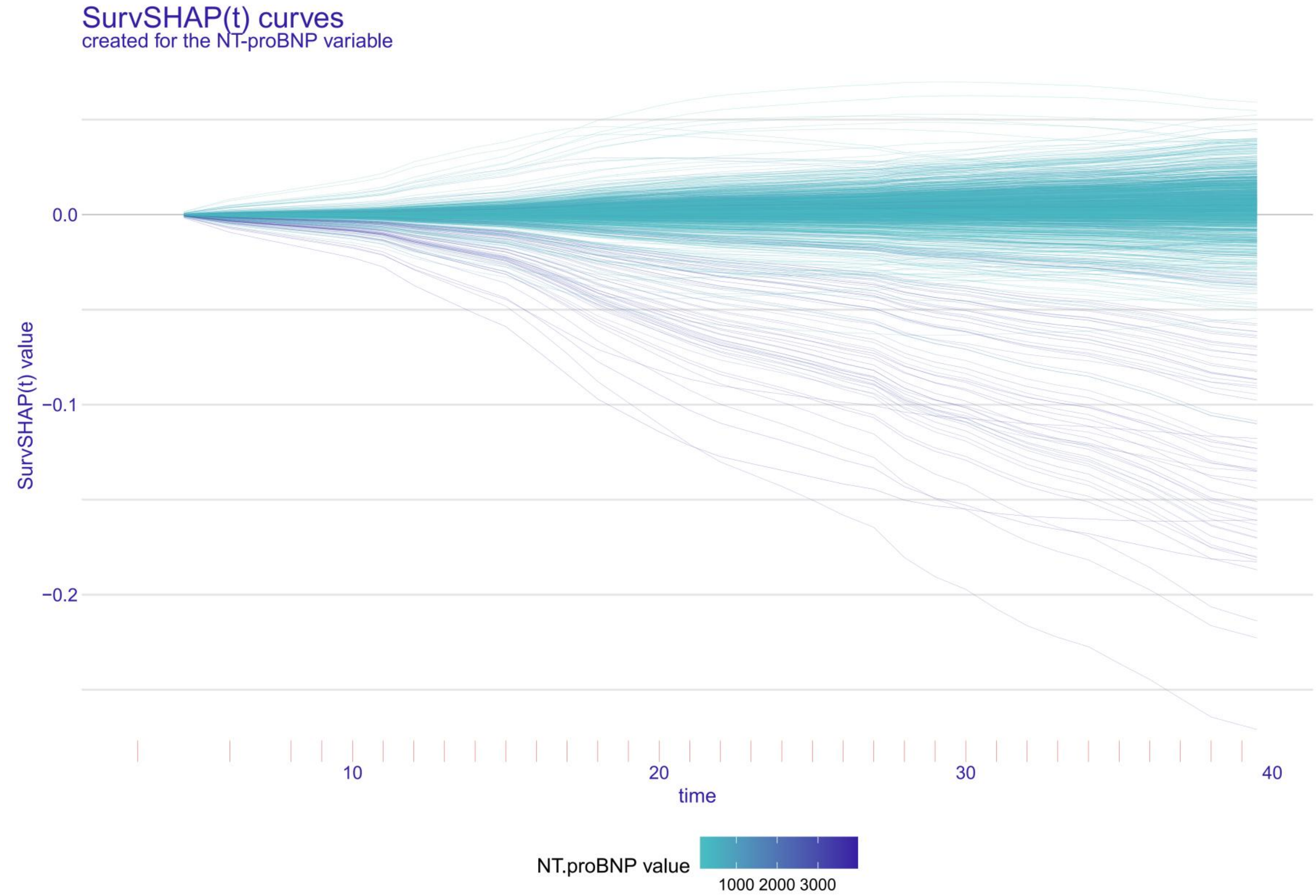
SurvSHAP(t) curves
created for the NT-proBNP variable
0.0
−0.1
−0.2
SurvSHAP(t) value
10
20
30
40
time
NT.proBNP value
1000 2000 3000

Figure S8. Local interpretation of individual feature contributions to MACE risk for a single patient with no MACE events within 40 months. (A) The plot shows the impact of feature variables on the patient's MACE risk, illustrating whether their values increase or decrease the likelihood of survival. (B) The plot compares predictions from the black-box model with those from the surrogate model. (C) SurvSHAP(t) quantifies the contribution of each feature to the model's prediction for an individual patient.

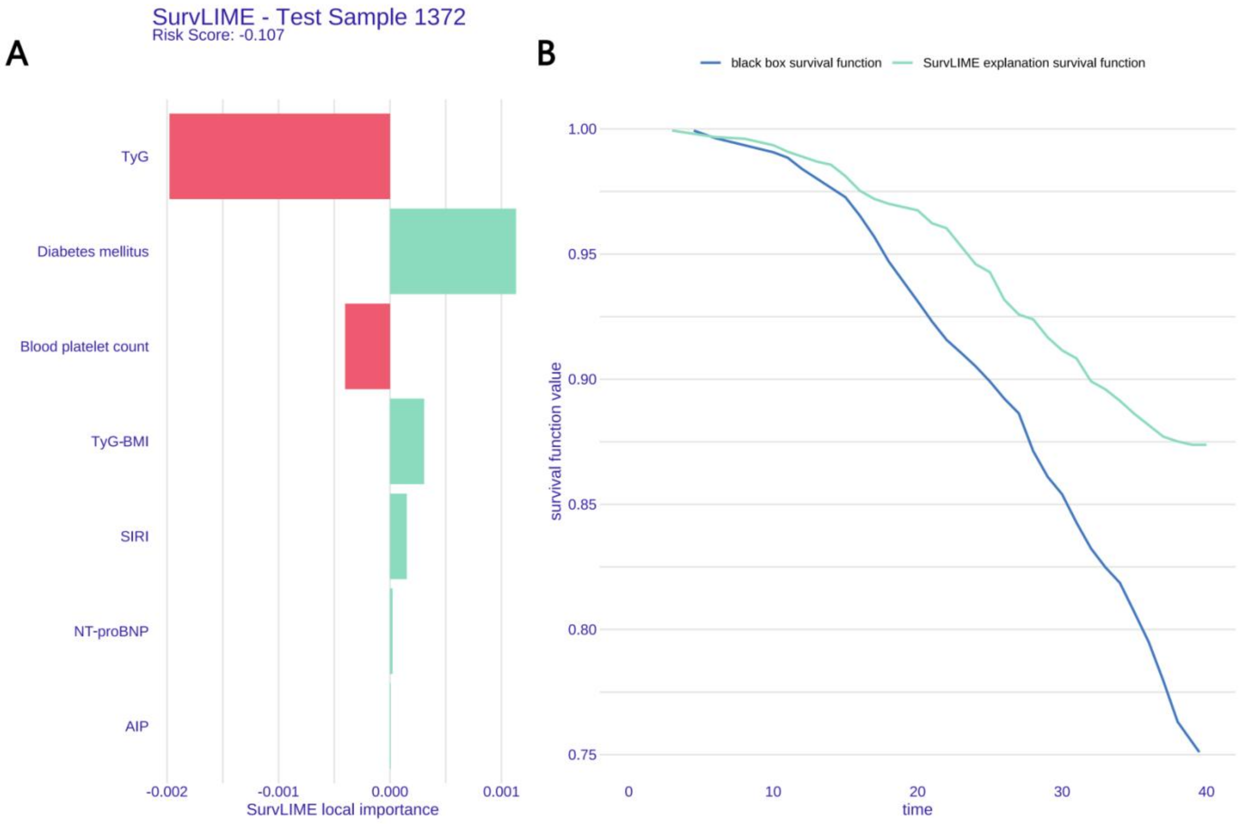

C

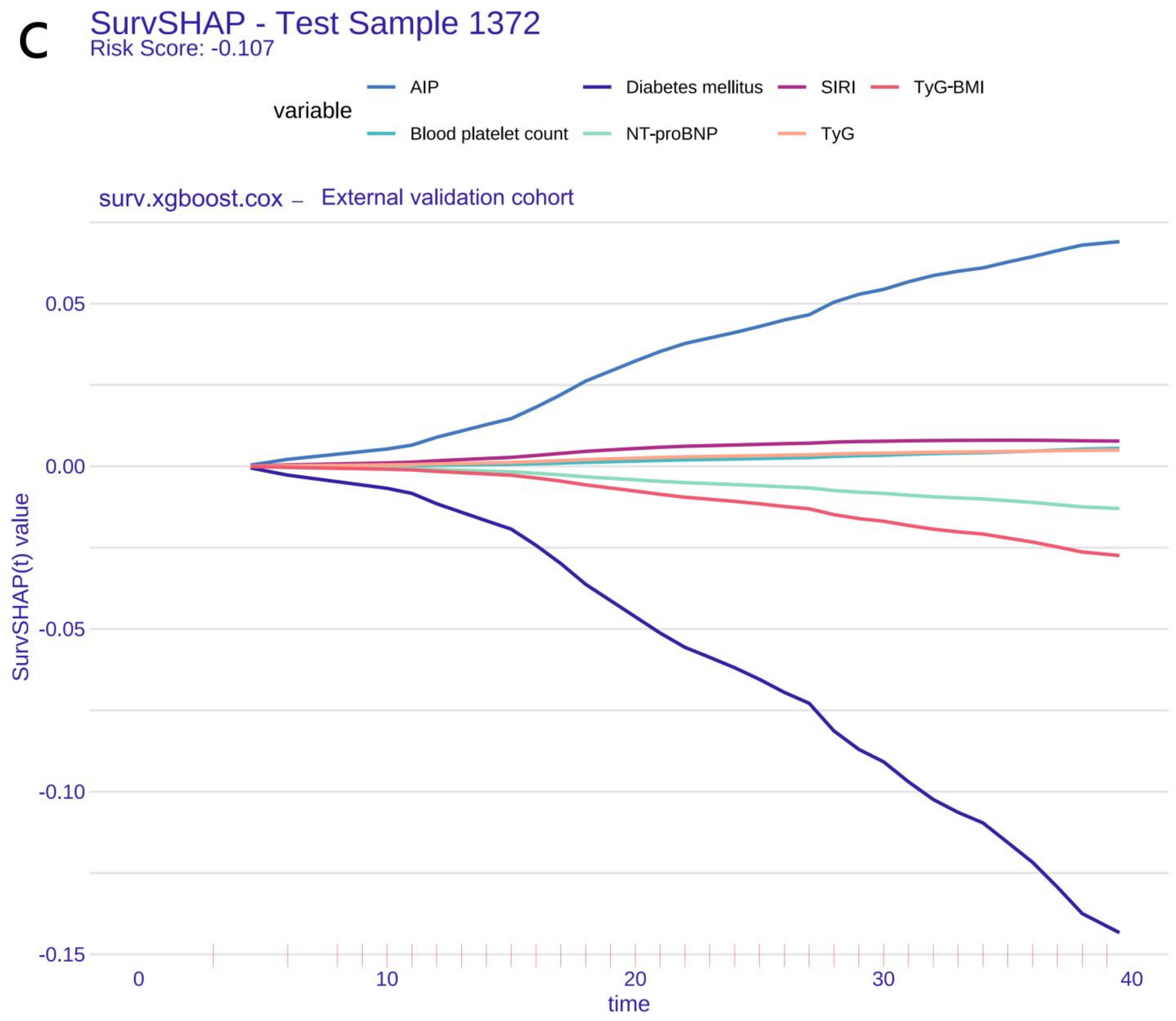

SurvSHAP - Test Sample 1372
Risk Score: -0.107
variable
AIP
Diabetes mellitus
SIRI
TyG-BMI
Blood platelet count
NT-proBNP
TyG
surv.xgboost.cox – External validation cohort
0.05
0.00
-0.05
-0.10
-0.15
SurvSHAP(t) value
0
10
20
30
40
time

Figure S9. The interactive dashboard calculates the predicted incidence of MACEs for individual UA-HFpEF patients.

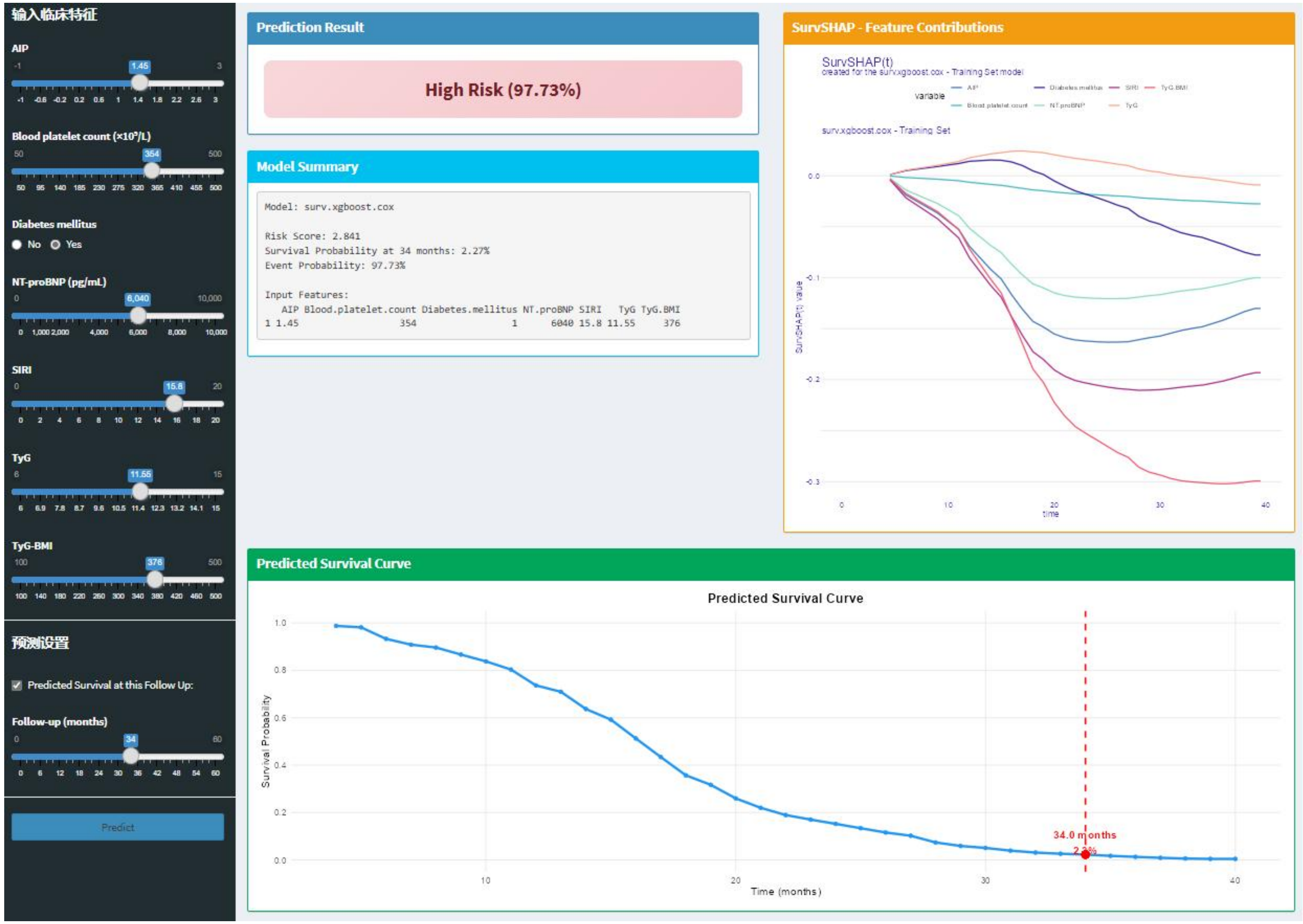

Supplement: Multimedia Appendix 1 [file jmir-v27-e78402-s001.pdf]
